# Associations of Human Milk Oligosaccharides With Otitis Media and Lower and Upper Respiratory Tract Infections up to 2 Years: The Ulm SPATZ Health Study

**DOI:** 10.3389/fnut.2021.761129

**Published:** 2021-10-25

**Authors:** Linda P. Siziba, Marko Mank, Bernd Stahl, Deborah Kurz, John Gonsalves, Bernadet Blijenberg, Dietrich Rothenbacher, Jon Genuneit

**Affiliations:** ^1^Department of Pediatrics, Pediatric Epidemiology, Medical Faculty, Leipzig University, Leipzig, Germany; ^2^Danone Nutricia Research, Utrecht, Netherlands; ^3^Department of Chemical Biology & Drug Discovery, Utrecht Institute for Pharmaceutical Sciences, Utrecht University, Utrecht, Netherlands; ^4^Institute of Epidemiology and Medical Biometry, Ulm University, Ulm, Germany

**Keywords:** human milk oligosaccharides (HMOs), otitis media (OM), lower respiratory tract infections (LRTI), upper respiratory tract infections (URTI), human milk groups, targeted LC-MS/MS^2^

## Abstract

**Background:** Human milk oligosaccharides (HMOs) support and concurrently shape the neonatal immune system through various mechanisms. Thereby, they may contribute to lower incidence of infections in infants. However, there is limited evidence on the role of individual HMOs in the risk of otitis media (OM), as well as lower and upper respiratory tract infections (LRTI and URTI, respectively) in children up to 2 years.

**Objective:** To investigate whether individual HMO concentrations measured at 6 weeks of lactation were associated with risk of OM, LRTI or URTI up to 2 years in breastfed infants. Associations with OM, LRTI and URTI were determined for the most prominent human milk oligosaccharides including 13 neutral, partly isomeric structures (trioses up to hexaoses), two acidic trioses, and lactose.

**Design:** HMO measurements and physician reported data on infections were available from human milk samples collected at 6 weeks postpartum (*n* = 667). Associations of HMOs with infections were assessed in crude and adjusted models using modified Poisson regression.

**Results:** Absolute concentrations (median [min, max], in g/L) of 2′-fucosyllactose (2′-FL) tended (*p* = 0.04) to be lower, while lacto-N-tetraose (LNT) was higher in the milk for infants with OM in the 1st year of life (*p* = 0.0046). In the milk of secretor mothers, LNT was significantly higher in the milk for infants with OM (RR [95% CI]: 0.98 [0.15, 2.60]) compared to infants without OM (RR [95% CI]: 0.76 [0.14, 2.90]) at 1 year (*p* = 0.0019). No statistically significant milk group differences and associations were observed for OM, LRTI, and URTI (*p* > 0.0031).

**Conclusion:** Our findings suggest that neither prominent neutral individual HMOs (ranging from 2′-FL to LNDFHs) nor acidic human milk sialyllactoses or lactose are significantly associated with a reduced or increased risk of infections in infants up to 2 years of age. Further research is needed to determine whether specific HMOs could potentially reduce the incidence or alleviate the course of distinct infections in early life.

## Introduction

Otitis media (OM) is a common infectious disease during infancy, which is also responsible for a huge burden of disease in both emerging and established economies (1). In addition, respiratory infections are one of the leading causes of morbidity in infants and young children (2, 3). Recent reviews (4, 5) highlighted several studies that have demonstrated a protective effect of breastfeeding on OM and respiratory infections in children up to 2 years of age. Hence, human milk is regarded important as an early life exposure for the development of a healthy immune system (6–8). Of note, human milk comprises several multifunctional components which function as chemokines, antimicrobial proteins or peptides, antioxidants, growth factors, anti-inflammatory elements, prebiotics, enzymes, probiotics, and nutrients for the infant (9, 10). However, very little is known about the nature of the relationship or interactions, if any, between these multiple components that are present in human milk, and their impact on the infant (11, 12).

Of interesting significance are human milk oligosaccharides (HMOs), important constituents of human milk that seem to have anti-microbial and anti-viral effects which could potentially contribute to lower incidence of respiratory and other infections in breastfed infants (13–17). Although the structures and quantities of HMOs differ significantly amongst women and both are dependent on maternal secretor and Lewis blood group status (18, 19), their influence on the infant's microbiome and immune maturation is well documented (14, 20–23).

However, the mechanism through which individual HMOs act as an antiviral is based on *in vitro* and *ex vivo* studies (24–27). Many viruses, bacterial pathogens or toxins need to adhere to mucosal surfaces to colonise or invade the host and cause disease (22). The biological structure of HMOs is similar to all cell surface glycan receptors and serves as decoy receptors that block the pathogens from binding to epithelial cells (20, 21). Consequently, the decoy receptor bound pathogens are then unable to attach to the cell surfaces and are excreted without causing disease. In addition, HMOs also modulate differentiation and death of intestinal cells which potentially impacts the infant's susceptibility to infections (28). As such, both neutral and acidic HMOs have been shown to exhibit anti-effective properties. For instance, some individual HMO structured have been associated with protection against diarrhoea in infants (29), postnatal HIV-transmission during breastfeeding (30), necrotizing enteritis (NEC) (31, 32) and reducing rotavirus infections (22), amongst others. As a result, HMOs play a key role in shaping the infant gut microbiome, controlling enteric infections, and protecting the newborn infant, plausibly in combination with other bioactive components in human milk, including non-immunoglobulin proteins and milk fat globule membrane.

In spite of the substantial interest in this area of research, the evidence is still scarce. We are only aware of two other studies that investigated associations of HMOs with OM and upper respiratory tract infections (URTI) (33) and acute respiratory infections (34). The outcomes among breastfed children in both studies were assessed at 6 and 24 weeks of age (33), and at 6–7 months of age (34). Still, the sample size for one study (33) was limited (*n* = 49) and human milk samples were collected only at 2 weeks (33) and 6 months (34) postpartum. We previously showed a significant relationship of time, maternal secretor and milk group status on HMOs across lactation (35). Based on this, the HMO structures present and supplied to the infant through human milk will differ significantly. Maternal secretor and milk group status should therefore be considered when investigating clinical health outcomes in infants. Yet, maternal secretor status was only accounted for in one recent study (34).

Furthermore, Stepans et al. (33) investigated one specific HMO, LNFP II (lacto-*N*-fucopentaose II), while Jorgensen et al. (34) investigated several other bioactive proteins in addition to 51 individual HMOs and structure specific groups of HMOs. In light of this, a broader understanding of the potential effects of HMOs in increasing or reducing susceptibility to infections in breastfed infants is warranted. Thus, to contribute to this area of research, we not only measured lactose and several individual HMOs (~15) using more sensitive and robust techniques (35), but we also took into account maternal secretor status and milk groups. The aim of this study was to investigate the associations of individual HMOs measured at 6 weeks of lactation with OM, and with lower and upper respiratory tract infections (LRTI and URTI, respectively) in the first and second year of life in the context of a large birth cohort study.

## Materials and Methods

### Study Design and Population

Data were obtained from the Ulm SPATZ Health Study, an ongoing birth cohort study, in which a total of 970 mothers (49% of all eligible families) and their 1,006 newborn infants were recruited shortly after their delivery, during their hospital stay at the University Medical Centre Ulm, southern Germany, between April 2012 and May 2013 (36). Of note, University Medical Centre was the only hospital in Ulm and surrounding areas, thus we have a fairly representative sample of the general population. Inadequate German language skills, outpatient childbirth, maternal age <18 years, postpartum transfer of mother or child to intensive care unit, or stillbirth were all reasons for exclusion from participating in the study. All participants signed written informed consent prior to the study and participation in the study was completely voluntary. Ethical approval for the SPATZ study was attained from the Ethics board of Ulm University (No. 311/11).

### Data Collection and Measurements

Demographic, lifestyle and birth-related data including but not limited to child sex, delivery mode, maternal age, level of education, parity, and pre-pregnancy body mass index (BMI calculated as [mass(kg)/height(m)^2^]) were collected using self-administered questionnaires, electronic hospital charts, and routine screening examinations. Questionnaires documented social demographic information, living situation and lifestyle factors. Further clinical data related to child's delivery and the mother's pregnancy were obtained from routine paper documentation. Mothers were asked if the child was still getting human milk at the time of sample collection and if the mother had introduced any complementary foods or liquids in addition to human milk. When the mother stated that no further liquid, semi-solid or solid food had been given to the child up to that point, this was classified as exclusive breastfeeding. Subsequently, a more definitive category of exclusive breastfeeding was then derived, based on maternal recall of introduction of other liquids or foods, at each time point. Reported doctor's diagnoses of OM, LRTI (including pneumonia, bronchitis, pertussis, tracheobronchitis, Krupp, bronchiolitis and flu), and URTI (including rhinitis, pharyngitis, tonsillitis and epiglottitis) were assessed at 1 and 2 years of age, for the first and the second year of life, respectively, by standardised, self-administered questionnaires from the children's primary care paediatricians. Several other health outcomes were also similarly assessed concurrently using these questionnaires. Additional data was collected at 6 weeks, 6 months, and 12 months after delivery by telephone interview or self-administered questionnaires sent by post if participants were not reachable by telephone of had previously stated breastfeeding cessation. Subsequent follow-ups were done yearly, and follow-ups is still on going.

Human milk samples were collected at approximately 6 weeks [Mean (SD), 5.9 (0.7) weeks] post-delivery from willing lactating mothers who were still breastfeeding at the time of sample collection. Lactating mothers were instructed to clean the breast and manually express or pump human milk between 9 am and 12 pm, after breakfast and before lunch, but at least 1 h after the infant's last feed. Where necessary, trained study nurses helped mothers with expression of human milk. Human milk samples were stored in the refrigerator by the mothers until study nurses collected them from their homes on the same day if milk was expressed between 9 am and 12 pm (76.9%), or on the next day if milk was expressed in the evening [after 12 pm, 9%; and before 9 am, 13.8%] and delivered them refrigerated to the study centre. Following which, they were separated into aliquots and frozen within 48 h and kept until further analysis.

### Analysis of HMOs

Human milk samples were stored at −80°C until analysis of HMOs in 2019 as previously described (35). Briefly, individual native HMOs were identified and quantified using targeted liquid chromatography electrospray ionisation tandem mass spectrometry (LC-ESI-MS^2^) in negative ion mode. Quantification of absolute HMO concentrations was done for lactose and 16 of the most abundant HMOs comprising: 2′-fucosyllactose (2′-FL); 3-fucosyllactose (3-FL); 3′-sialyllactose (3′-SL); 4′-galactosyllactose (4′-GL); 6′-galactosyllactose (6′-GL); 3,2′-difucosyllactose (DFL); 6′-sialyllactose (6′-SL); lacto-N-tetraose (LNT); lacto-N-neotetraose (LNnT); lacto-N-fucopentaose I (LNFP I); lacto-N-fucopentaose II (LNFP II); lacto-N-fucopentaose III (LNFP III); lacto-N-fucopentaose V (LNFP V); lacto-N-difucohexaose I (LNDFH I); and the sum of lacto-N-difucohexaose II and lacto-N-neodifucohexaose II (LNDFH II + LNnDFH II, standard containing both). Milk group and secretor status in this study were determined as previously described (35), based on the presence or absence of α1,2- and α1,4-fucosylated HMOs (Table 1). We essentially used the concentrations of LNFP I, LNFP II, and LNDFH I as proxies for FUT2 and FUT3 activities. Thus, human milk samples which contained both α1,2- and α1,4-fucosylated HMOs, like LNDFH I, were attributed to group I. Group II milk comprised human milk samples in which LNFP I and LNDFH I were absent [i.e., below the limit of quantification (< LLOQ)]. Group III milk comprised samples in which LNFP II and LNDFH I were not present (< LLOQ) and group IV comprised samples which did not present LNFP I and LNFP II (< LLOQ). Human milk samples attributed to group I and II were then grouped as secretors, while group II and IV milk were grouped as non-secretors. For the purpose of this study, HMO concentrations measured at 6 weeks were used to determine associations with OM, LRTI and URTI in the first and second years of the infant's life, respectively. Human milk samples with a complete set of HMO data and outcomes on infections (either one, in the first or second year) were available from up to 667 mothers.

**Table 1:** Basis on which human milk samples were classified to the different milk groups (types).

**Milk type**	**Condition**
I	If not Type II, III or IV
II	LNFP I & LNDFH I < LLOQ
III	LNFP II & LNDFH I < LLOQ
IV	LNFP I & II < LLOQ

*LNFP I, lacto-N-fucopentaose-I; LNFP II, lacto-N-fucopentaose-II; LNDFH I, lacto-N-difucohexaose I; < LLOQ, below the limit of quantification*.

### Statistical Analysis

Data for 4′-GL were excluded because >90% of values were found to be below the lower limit of quantification (< LLOQ). For the remaining HMOs, all values < LLOQ were replaced by LLOQ / √(2) and the values higher than the upper limit of quantification (>ULOQ) were extrapolated for 3-FL, 3′-SL, DFL, 6′-GL, 6′-SL, LNT, LNFP I, LNFP II, LNDFH I, LNDFH II + LNnDFH II (35). Absolute HMO concentrations (g/L) measured at 6 weeks of lactation stratified by OM, LRTI or UTRI in the first or second year of life are presented as mean (SD) and median [min, max]. An unpaired two samples Wilcoxon sum rank test was used to evaluate differences of HMO and lactose concentrations in the milk for children with or without OM, LRTI or URTI. Modified Poisson regression analyses (37) were used to determine associations between individual HMOs and lactose with OM, LRTI or URTI in the first or second year of life, respectively in crude and in adjusted models. Risk ratios (RR) and their 95% confidence intervals (95% CI) were obtained by exponentiation of the observed coefficients, as a logarithmic link function was used to model the HMO concentrations as continuous independent variables. Models were adjusted for infant sex, maternal allergy, exclusive breastfeeding at 6 weeks of lactation, delivery mode, parity, secretor status and milk group. These covariates were selected to be included in the models based on their influence on HMOs (35, 38–40). A stratified analysis was done to investigate these associations amongst infants receiving secretor or non-secretor milk and those receiving milk attributed to milk group I or II. A Bonferroni-adjusted threshold of α= 0.05/16 = 0.0031 was used as a level of statistical significance following correction for multiple testing. All statistical analyses were performed with R (version 3.5.1; R Foundation for Statistical Computing).

## Results

A total of 667 (68.8% of women participating in SPATZ) lactating women had HMO and infant data on either OM, LRTI or URTI in the first or second year of life (Table 2). The majority (75%) of infants were receiving human milk exclusively at 6 weeks. More than 70% of the infants had a report of at least one episode of OM in the first or second year of life. Almost a third (30%) had a positive report of LRTI in the first or second year of life. Similarly, more than half (60%) reported URTI in the first or second year of life.

**Table 2:** Characteristics of lactating mothers and infants who had a complete set of data on human milk oligosaccharides and atopic dermatitis available in the Ulm SPATZ Health Study.

		**6 weeks samples (*n* = 667)**
Mother		*n* (%)
	Age (years)	33.05 (4.58)^a^
Age category (years)		
	<30	30 (4.5)
	30–35	467 (70.1)
	>35	169 (25.4)
BMI category 6 weeks		
	Underweight (BMI <18.5 kg/m^2^)	6 (1.0)
	Normal (18.5 ≤ BMI <25 kg/m^2^)	345 (57.9)
	Overweight (25 ≤ BMI <30 kg/m^2^)	179 (30.0)
	Obese (BMI ≥ 30·00 kg/m^2^)	66 (11.1)
Parity (*n* births of foetus ≥ 24 weeks)		
	0 births	343 (51.5)
	≥1 birth	323 (48.5)
Milk group		
	I	493 (73.9)
	II	119 (17.8)
	II	47 (7.0)
	IV	8 (1.2)
Maternal allergy		
	Yes	203 (30.6)
	No	461 (69.4)
**Infant**		
	Female	316 (47.4)
	Male	351 (52.6)
Gestation category		
	≤36 weeks	183 (27.4)
	Between 36 and 41 weeks	382 (57.3)
	≥41 weeks	102 (15.3)
Delivery mode		
	Vaginal spontaneous	459 (68.9)
	Elective caesarean	66 (9.9)
	Emergency caesarean	80 (12.0)
	Vaginal assisted	61 (9.2)
Exclusive breastfeeding at 6 weeks		
	Yes	503 (75.4)
	No	164 (24.6)
Otitis media in the first year of life		
	Yes	440 (89.8)
	No	50 (10.2)
Otitis media in the second year of life		
	Yes	363 (72.0)
	No	141 (28.0)
LRTI in the first year of life		
	Yes	146 (29.6)
	No	348 (70.4)
LRTI in the second year of life		
	Yes	240 (44.6)
	No	298 (55.4)
URTI in the first year of life		
	Yes	327 (66.2)
	No	167 (33.8)
URTI in the second year of life		
	Yes	433 (80.9)
	No	102 (19.1)

a *Mean (SD). SD, Standard deviation; BMI, Body mass index; LRTI, Lower respiratory tract infections; URTI, Upper respiratory tract infections*.

Although there were some differences in absolute HMO concentrations (g/L) in the milk for infants with OM in the first or second year of life, these differences were not statistically significant (Bonferroni correction α threshold = 0.0031). However, at the conventional level of significance (*p* < 0.05), 2′-FL was (Wilcoxon sum-rank test, *p* = 0.04) lower while LNT was considerably higher in the human milk for infants with OM at 1 year (*p* = 0.0046, Table 3). However, after Bonferroni correction, the differences in absolute HMO concentrations (g/L) in the human milk for infants who developed OM in the first or second year of life, were not statistically significant (Bonferroni correction α threshold = 0.0031).

**Table 3:** Absolute human milk oligosaccharide concentrations (g/L) measured at 6 weeks and otitis media in infants in the first or second year of life in the Ulm SPATZ Health Study.

	**OM in the first year of life**			**OM in the second year of life**		
	**OM Yes (*n* = 50)**	**OM No (*n* = 440)**	***p***	**OM Yes (*n* = 141)**	**OM No (*n* = 363)**	***p***
Lactose						
Mean (SD)	66.8 (4.31)	66.6 (3.69)		66.4 (4.04)	66.7 (3.68)	
Median [min, max]	67.0 [58.0, 74.0]	67.0 [52.0, 77.0]	0.64	67.0 [56.0, 77.0]	67.0 [52.0, 74.0]	0.42
2′-FL						
Mean (SD)	1.93 (1.32)	2.31 (1.41)		2.26 (1.34)	2.29 (1.43)	
Median [min, max]	2.00 [0.13, 6.30]	2.50 [0.13, 6.60]	0.04	2.50 [0.13, 6.30]	2.50 [0.13, 6.60]	0.95
3-FL						
Mean (SD)	0.71 (0.55)	0.67 (0.54)		0.61 (0.51)	0.68 (0.55)	
Median [min, max]	0.55 [0.11, 2.10]	0.49 [0.03, 2.90]	0.65	0.42 [0.03, 2.90]	0.51 [0.03, 2.60]	0.12
3′-SL						
Mean (SD)	0.16 (0.05)	0.15 (0.04)		0.15 (0.05)	0.15 (0.04)	
Median [min, max]	0.15 [0.08, 0.27]	0.15 [0.05, 0.32]	0.64	0.15 [0.05, 0.30]	0.15 [0.07, 0.32]	0.79
6′-GL						
Mean (SD)	0.02 (0.02)	0.02 (0.01)		0.02 (0.01)	0.02 (0.01)	
Median [min, max]	0.02 [0.01, 0.15]	0.02 [0.004, 0.09]	0.67	0.02 [0.01, 0.15]	0.02 [0.004, 0.09]	0.51
DFL						
Mean (SD)	0.16 (0.15)	0.18 (0.17)		0.17 (0.14)	0.18 (0.18)	
Median [min, max]	0.15 [0.01, 0.72]	0.18 [0.01, 1.80]	0.11	0.15 [0.01, 0.72]	0.18 [0.01, 1.80]	0.56
6′-SL						
Mean (SD)	0.25 (0.09)	0.26 (0.11)		0.25 (0.10)	0.26 (0.11)	
Median [min, max]	0.24 [0.07, 0.54]	0.24 [0.06, 0.73]	0.84	0.23 [0.07, 0.54]	0.24 [0.06, 0.73]	0.33
LNT						
Mean (SD)	1.09 (0.50)	0.92 (0.49)		0.98 (0.53)	0.92 (0.48)	
Median [min, max]	0.99 [0.15, 2.60]	0.84 [0.14, 3.10]	0.0046	0.86 [0.15, 3.10]	0.84 [0.14, 2.90]	0.31
LNnT						
Mean (SD)	0.08 (0.05)	0.08 (0.06)		0.09 (0.06)	0.08 (0.06)	
Median [min, max]	0.07 [0.01, 0.26]	0.07 [0.01, 0.34]	0.50	0.07 [0.01, 0.34]	0.07 [0.01, 0.33]	0.66
LNFP I						
Mean (SD)	0.50 (0.48)	0.48 (0.50)		0.53 (0.48)	0.48 (0.46)	
Median [min, max]	0.35 [0.04, 2.20]	0.38 [0.04, 3.00]	0.97	0.42 [0.04, 2.70]	0.36 [0.04, 3.00]	0.33
LNFP V						
Mean (SD)	0.04 (0.04)	0.04 (0.04)		0.04 (0.04)	0.04 (0.04)	
Median [min, max]	0.03 [0.01, 0.17]	0.02 [0.01, 0.24]	0.08	0.02 [0.01, 0.17]	0.02 [0.01, 0.24]	0.54
LNFP III						
Mean (SD)	0.190 (0.07)	0.18 (0.07)		0.18 (0.07)	0.18 (0.07)	
Median [min, max]	0.19 [0.06, 0.43]	0.18 [0.04, 0.48]	0.31	0.17 [0.05, 0.43]	0.18 [0.04, 0.48]	0.45
LNFP II						
Mean (SD)	0.41 (0.42)	0.34 (0.40)		0.32 (0.37)	0.35 (0.41)	
Median [min, max]	0.22 [0.04, 1.60]	0.17 [0.04, 2.50]	0.23	0.15 [0.04, 1.60]	0.18 [0.04, 2.50]	0.25
LNDFH I						
Mean (SD)	0.50 (0.37)	0.53 (0.39)		0.53 (0.38)	0.52 (0.39)	
Median [min, max]	0.55 [0.02, 1.50]	0.58 [0.02, 1.90]	0.58	0.55 [0.02, 1.70]	0.58 [0.02, 1.90]	0.88
LNDFH II + LNnDFH II						
Mean (SD)	0.08 (0.12)	0.06 (0.11)		0.06 (0.10)	0.07 (0.11)	
Median [min, max]	0.02 [0.01, 0.46]	0.02 [0.01, 0.73]	0.35	0.01 [0.01, 0.46]	0.02 [0.01, 0.73]	0.28
Sum of HMOs						
Mean (SD)	6.12 (1.29)	6.23 (1.29)		6.19 (1.33)	6.24 (1.29)	
Median [min, max]	6.18 [3.70, 9.69]	6.16 [2.41, 11.6]	0.53	6.18 [2.41, 9.80]	6.17 [2.76, 11.6]	0.79

*p values derived from Wilcoxon sum-rank test comparing HMO concentrations between infants with and without otitis media at 1 or 2 years. Bonferroni-adjusted level of statistical significance is α = 0.05/16 = 0.0031*.

*OM, Otitis media; RR, Risk Ratio; CI, Confidence intervals; HMO, human milk oligosaccharides*.

*2′-FL, 2′-fucosyllactose; 3-FL, 3-fucosyllactose; 3′-SL, 3′-sialyllactose; 6′-GL, 6′-galactosyllactose; DFL, 3,2′-difucosyllactose; 6′-SL, 6′-sialyllactose; LNT, lacto-N-tetraose; LNnT, lacto-N-neotetraose; LNFP I, lacto-N-fucopentaose-I; LNFP V, lacto-N-fucopentaose-V; LNFP III, lacto-N-fucopentaose-III; LNFP II, lacto-N-fucopentaose-II; LNDFH I, lacto-N-difucohexaose I; LNDFH II, lacto-N-difucohexaose II; LNnDFH II, lacto-N-neodifucohexaose II*.

A similar pattern of seemingly lower 2′-FL (Median [min, max)], although not statistically significant following correction for multiple testing, was evident in the secretor milk of infants with OM (2.40 [0.36, 6.30] g/L) compared to those without OM (2.70 [0.13, 6.60] g/L) in the first year of life (*p* = 0.009, Supplementary Table 1). Amongst infants receiving secretor milk, absolute concentrations of LNT were significantly higher in the milk for infants with OM (0.98 [0.15, 2.60] (g/L), *p* = 0.0019) compared to infants without OM (0.76 [0.14, 2.90] g/L) in the first year of life (Supplementary Table 1). This statistically significant difference in LNT concentrations was not evident for OM in the second year of life (Supplementary Table 2).

At the conventional level of significance (*p* < 0.05), absolute concentrations of LNT were higher in group I milk of infants with OM (0.98 [0.15, 2.60] g/L) compared to their counterparts (0.77 [0.14, 2.90] g/L, *p* = 0.0067, Supplementary Table 3) but not in the second year of life (*p* = 0.28, Supplementary Table 4). There were no statistically significant differences (i.e., at the conventional and Bonferroni corrected levels of significance) of individual HMOs in the milk for infants with or without LRTI (Supplementary Tables 5–9). Likewise, absolute concentrations of LNFP I (0.36 [0.04, 3.00] g/L) and total HMOs (6.10 [2.41, 11.6] g/L) were lower (*p* < 0.05) in the milk of infants with URTI in the first year compared to their counterparts (0.48 [0.04, 2.70], and 6.60 [3.39, 9.80] g/L, respectively, Supplementary Table 10) in the second year of life. This pattern was not present in secretor milk of infants with URTI in the first year (Supplementary Table 11) but in the second year of life (Supplementary Table 12). This was also the case when human milk samples were stratified by milk group (Supplementary Tables 13, 14).

Following Bonferroni correction (α = 0.0031), there were no statistically significant associations of individual HMOs with the risk of OM in the first or second year of life (Table 4). However, there were some associations that were statistically significant at the conventional level (using α = 0.05). For example, higher LNT and higher LNFP V were both associated with an increased risk of OM in the first year [RR (95% CI): 1.17 (1.02, 1.34) and RR (95% CI): 1.16 (1.01, 1.35), respectively]. Higher LNT [RR (95% CI)] in secretor milk was significantly (*p* < 0.0031) associated with a higher risk [RR (95% CI): 1.27 (1.09, 1.49), *p* = 0.0025] of OM in the first year of life in crude models (not shown). This association was not statistically significant following adjustment for infant sex, maternal allergy, delivery mode, exclusive breastfeeding, parity and milk group [RR (95% CI): 1.25 (1.06, 1.47), *p* = 0.01; Table 5].

**Table 4:** Adjusted associations between human milk oligosaccharides measured at 6 weeks of lactation with otitis media in the first or second year of life in the Ulm SPATZ Health Study.

	**OM in the first year of life**			**OM in the second year of life**		
	**RR**	**95% CI**	***p***	**RR**	**95% CI**	***p***
Lactose	1.00	(0.98, 1.02)	0.96	1.00	(0.99, 1.01)	0.67
2′-FL	0.87	(0.76, 1.01)	0.06	0.97	(0.91, 1.05)	0.49
3-FL	1.03	(0.90, 1.19)	0.64	0.91	(0.82, 1.02)	0.11
3′-SL	1.02	(0.93, 1.11)	0.71	1.01	(0.96, 1.07)	0.71
6′-GL	1.19	(0.92, 1.54)	0.18	1.09	(0.96, 1.25)	0.19
DFL	0.88	(0.70, 1.10)	0.25	0.93	(0.81, 1.07)	0.30
6′-SL	0.99	(0.89, 1.10)	0.89	0.95	(0.88, 1.03)	0.23
LNT	1.17	(1.02, 1.34)	0.03	1.07	(0.98, 1.18)	0.14
LNnT	0.92	(0.77, 1.09)	0.33	1.01	(0.89, 1.14)	0.90
LNFP I	1.07	(0.84, 1.37)	0.60	1.09	(0.93, 1.27)	0.30
LNFP V	1.16	(1.01, 1.35)	0.04	1.03	(0.91, 1.16)	0.66
LNFP III	1.05	(0.95, 1.16)	0.31	0.98	(0.90, 1.06)	0.55
LNFP II	1.14	(0.96, 1.36)	0.12	0.95	(0.82, 1.09)	0.47
LNDFH I	0.96	(0.82, 1.12)	0.58	1.00	(0.90, 1.11)	0.99
LNDFH II + LNnDFH II	1.17	(0.91, 1.50)	0.21	0.90	(0.74, 1.11)	0.35
Total HMOs	0.99	(0.95, 1.05)	0.79	0.99	(0.95, 1.03)	0.62

*Associations determined by modified Poisson regression. Models adjusted for infant sex, maternal allergy, delivery mode, exclusive breastfeeding, parity, secretor status and milk group. Bonferroni-adjusted level of statistical significance is α= 0.05/16 = 0.0031*.

*OM, Otitis media; RR, Risk Ratio; CI, Confidence intervals; HMO, human milk oligosaccharides*.

*2′-FL, 2′-fucosyllactose; 3-FL, 3-fucosyllactose; 3′-SL, 3′-sialyllactose; 6′-GL, 6′-galactosyllactose; DFL, 3,2′-difucosyllactose; 6′-SL; 6′-sialyllactose; LNT, lacto-N-tetraose; LNnT, lacto-N-neotetraose; LNFP I, lacto-N-fucopentaose-I; LNFP V, lacto-N-fucopentaose-V; LNFP III, lacto-N-fucopentaose-III; LNFP II, lacto-N-fucopentaose-II; LNDFH I, lacto-N-difucohexaose I; LNDFH II, lacto-N-difucohexaose II; LNnDFH II, lacto-N-neodifucohexaose II*.

**Table 5:** Adjusted associations between human milk oligosaccharides in secretor milk measured at 6 weeks of lactation with otitis media in the first or second year of life in the Ulm SPATZ Health Study.

	**OM in the first year of life**			**OM in the second year of life**		
	**RR**	**95% CI**	***p***	**RR**	**95% CI**	***p***
Lactose	1.00	(0.98, 1.02)	0.77	1.00	(0.98, 1.01)	0.45
2′-FL	0.89	(0.77, 1.03)	0.12	0.98	(0.92, 1.05)	0.62
3-FL	0.98	(0.81, 1.19)	0.88	0.94	(0.83, 1.06)	0.30
3′-SL	1.02	(0.93, 1.12)	0.66	1.01	(0.95, 1.07)	0.76
6′-GL	0.98	(0.83, 1.17)	0.86	0.98	(0.88, 1.08)	0.67
DFL	0.85	(0.67, 1.06)	0.15	0.92	(0.80, 1.05)	0.21
6′-SL	1.00	(0.89, 1.13)	0.99	0.96	(0.88, 1.04)	0.33
LNT	1.25	(1.06, 1.47)	0.01	1.07	(0.96, 1.19)	0.23
LNnT	0.90	(0.74, 1.08)	0.26	0.99	(0.87, 1.12)	0.82
LNFP I	1.12	(0.88, 1.42)	0.35	1.11	(0.96, 1.29)	0.16
LNFP V	1.17	(0.94, 1.47)	0.16	1.02	(0.89, 1.17)	0.80
LNFP III	1.00	(0.88, 1.13)	0.95	0.94	(0.86, 1.03)	0.19
LNFP II	1.11	(0.83, 1.49)	0.47	0.99	(0.83, 1.18)	0.87
LNDFH I	0.91	(0.80, 1.05)	0.19	0.98	(0.90, 1.07)	0.66
LNDFH II + LNnDFH II	1.14	(0.84, 1.55)	0.39	0.98	(0.81, 1.19)	0.88
Total HMOs	0.98	(0.92, 1.06)	0.66	1.00	(0.96, 1.04)	0.90

*Associations determined by modified Poisson regression. Models adjusted for infant sex, maternal allergy, delivery mode, exclusive breastfeeding, parity and milk group. Bonferroni-adjusted level of statistical significance is α = 0.05/16 = 0.0031*.

*OM, Otitis media; RR, Risk Ratio; CI, Confidence intervals; HMO, human milk oligosaccharides*.

*2′-FL, 2′-fucosyllactose; 3-FL, 3-fucosyllactose; 3′-SL, 3′-sialyllactose; 6′-GL, 6′-galactosyllactose; DFL, 3,2′-difucosyllactose; 6′-SL, 6′-sialyllactose; LNT, lacto-N-tetraose; LNnT, lacto-N-neotetraose; LNFP I, lacto-N-fucopentaose-I; LNFP V, lacto-N-fucopentaose-V; LNFP III, lacto-N-fucopentaose-III; LNFP II, lacto-N-fucopentaose-II; LNDFH I, lacto-N-difucohexaose I; LNDFH II, lacto-N-difucohexaose II; LNnDFH II, lacto-N-neodifucohexaose II*.

Following Bonferroni correction, there were no further statistically significant associations between individual HMOs and the risk of OM, LRTI and URTI in the first or second year of life. Although, some associations were significant at conventional level (*p* < 0.05). For instance, higher 6′-GL in non-secretor milk was associated with a higher risk [RR (95% CI)] of OM in the first [1.95 (1.02, 3.73)] and second [1.61 (1.04, 2.50)] year of life (Table 6). Similarly, higher LNT in group I milk was associated with a higher risk [1.23 (1.04, 1.46)] of OM in the first year of life (Table 7). Higher 6′-GL in group II milk was also associated with a higher risk of OM in the first [1.96 (1.02, 3.74)] and second [1.60 (1.01, 2.55)] year of life (Table 8).

**Table 6:** Adjusted associations between human milk oligosaccharides in non-secretor milk measured at 6 weeks of lactation with otitis media in the first or second year of life in the Ulm SPATZ Health Study.

	**OM in the first year of life**			**OM in the second year of life**		
	**RR**	**95% CI**	***p***	**RR**	**95% CI**	***p***
Lactose	1.00	(0.96, 1.04)	0.88	1.01	(0.98, 1.03)	0.56
3-FL	0.98	(0.82, 1.18)	0.83	0.94	(0.81, 1.09)	0.42
3′-SL	0.99	(0.81, 1.20)	0.92	1.02	(0.89, 1.18)	0.78
6′-GL	1.95	(1.02, 3.73)	0.04	1.61	(1.04, 2.50)	0.03
6′-SL	0.97	(0.78, 1.20)	0.75	0.93	(0.80, 1.10)	0.40
LNT	0.98	(0.78, 1.24)	0.87	1.05	(0.87, 1.28)	0.62
LNnT	1.09	(0.78, 1.53)	0.62	1.27	(0.89, 1.81)	0.19
LNFP V	1.09	(0.91, 1.31)	0.33	1.07	(0.89, 1.29)	0.48
LNFP III	1.20	(0.99, 1.45)	0.07	1.10	(0.95, 1.26)	0.19
LNFP II	1.05	(0.87, 1.27)	0.60	1.00	(0.85, 1.18)	0.97
LNDFH II + LNnDFH II	1.09	(0.82, 1.46)	0.55	0.97	(0.77, 1.23)	0.81
Total HMOs	1.02	(0.91, 1.13)	0.78	1.00	(0.92, 1.09)	1.00

*Associations determined by modified Poisson regression. Models adjusted for infant sex, maternal allergy, delivery mode, exclusive breastfeeding, parity and milk group. Bonferroni-adjusted level of statistical significance is α = 0.05/16 = 0.0031*.

*OM, Otitis media; RR, Risk Ratio; CI, Confidence intervals; HMO, human milk oligosaccharides*.

*3-FL, 3-fucosyllactose; 3′-SL, 3′-sialyllactose; 6′-GL, 6′-galactosyllactose; 6′-SL; 6′-sialyllactose; LNT, lacto-N-tetraose; LNnT, lacto-N-neotetraose; LNFP V, lacto-N-fucopentaose-V; LNFP III, lacto-N-fucopentaose-III; LNFP II, lacto-N-fucopentaose-II; LNDFH II, lacto-N-difucohexaose II; LNnDFH II, lacto-N-neodifucohexaose II*.

**Table 7:** Adjusted associations between human milk oligosaccharides in group I milk measured at 6 weeks of lactation with otitis media in the first or second year of life in the Ulm SPATZ Health Study.

	**OM in the first year of life**			**OM in the second year of life**		
	**RR**	**95% CI**	***p***	**RR**	**95% CI**	***p***
Lactose	1.00	(0.98, 1.02)	0.69	1.00	(0.98, 1.01)	0.48
2′-FL	0.91	(0.78, 1.05)	0.20	0.99	(0.92, 1.06)	0.76
3-FL	0.98	(0.80, 1.19)	0.83	0.94	(0.83, 1.06)	0.30
3′-SL	1.02	(0.92, 1.13)	0.68	1.00	(0.95, 1.06)	0.92
6′-GL	0.96	(0.80, 1.15)	0.66	0.98	(0.88, 1.09)	0.65
DFL	0.83	(0.66, 1.05)	0.13	0.91	(0.79, 1.05)	0.21
6′-SL	1.01	(0.89, 1.14)	0.92	0.97	(0.89, 1.06)	0.52
LNT	1.23	(1.04, 1.46)	0.02	1.06	(0.95, 1.19)	0.28
LNnT	0.90	(0.74, 1.10)	0.30	0.99	(0.87, 1.13)	0.86
LNFP I	1.11	(0.87, 1.41)	0.39	1.14	(0.97, 1.32)	0.11
LNFP V	1.17	(0.93, 1.48)	0.18	1.02	(0.88, 1.18)	0.78
LNFP III	0.95	(0.85, 1.07)	0.43	0.94	(0.85, 1.03)	0.16
LNFP II	1.11	(0.83, 1.49)	0.48	0.99	(0.82, 1.18)	0.88
LNDFH I	0.91	(0.80, 1.05)	0.19	0.98	(0.90, 1.07)	0.65
LNDFH II + LNnDFH II	1.14	(0.84, 1.56)	0.40	0.99	(0.81, 1.20)	0.89
Total HMOs	0.99	(0.92, 1.05)	0.67	1.00	(0.96, 1.04)	0.95

*Associations determined by modified Poisson regression. Models adjusted for infant sex, maternal allergy, delivery mode, exclusive breastfeeding and parity. Bonferroni-adjusted level of statistical significance is α = 0.05/16 = 0.0031*.

*OM, Otitis media; RR, Risk Ratio; CI, Confidence intervals; HMO, human milk oligosaccharides*.

*2′-FL, 2′-fucosyllactose; 3-FL, 3-fucosyllactose; 3′-SL, 3′-sialyllactose; 6′-GL, 6′-galactosyllactose; DFL, 3,2′-difucosyllactose; 6′-SL, 6′-sialyllactose; LNT, lacto-N-tetraose; LNnT, lacto-N-neotetraose; LNFP I, lacto-N-fucopentaose-I; LNFP V, lacto-N-fucopentaose-V; LNFP III, lacto-N-fucopentaose-III; LNFP II, lacto-N-fucopentaose-II; LNDFH I, lacto-N-difucohexaose I; LNDFH II, lacto-N-difucohexaose II; LNnDFH II, lacto-N-neodifucohexaose II*.

**Table 8:** Adjusted associations between human milk oligosaccharides in group II milk measured at 6 weeks of lactation with otitis media in the first or second year of life in the Ulm SPATZ Health Study.

	**OM in the first year of life**			**OM in the second year of life**		
	**RR**	**Lower RR**	***P* value**	**RR**	**Lower RR**	***P* value**
Lactose	1.00	(0.96, 1.04)	0.90	1.00	(0.98, 1.03)	0.80
3-FL	0.98	(0.82, 1.18)	0.83	0.95	(0.82, 1.10)	0.46
3′-SL	0.99	(0.81, 1.21)	0.89	1.02	(0.88, 1.19)	0.74
6′-GL	1.96	(1.02, 3.74)	0.04	1.60	(1.01, 2.55)	0.05
6′-SL	0.97	(0.78, 1.20)	0.77	0.91	(0.78, 1.08)	0.27
LNT	0.98	(0.77, 1.24)	0.86	1.07	(0.87, 1.32)	0.50
LNnT	1.10	(0.78, 1.54)	0.60	1.24	(0.85, 1.82)	0.27
LNFP V	1.09	(0.91, 1.31)	0.35	1.12	(0.95, 1.33)	0.19
LNFP III	1.19	(0.99, 1.43)	0.06	1.13	(0.98, 1.31)	0.09
LNFP II	1.05	(0.87, 1.27)	0.60	1.00	(0.85, 1.18)	0.96
LNDFH II + LNnDFH II	1.09	(0.82, 1.46)	0.55	0.97	(0.77, 1.23)	0.82
Total HMOs	1.01	(0.91, 1.13)	0.79	1.01	(0.92, 1.10)	0.87

*Associations determined by modified Poisson regression. Models adjusted for infant sex, maternal allergy, delivery mode, exclusive breastfeeding and parity. Bonferroni-adjusted level of statistical significance is α = 0.05/16 = 0.0031*.

*OM, Otitis media; RR, Risk Ratio; CI, Confidence intervals; HMO, human milk oligosaccharides*.

*3-FL, 3-fucosyllactose; 3′-SL, 3′-sialyllactose; 6′-GL, 6′-galactosyllactose; 6′-SL, 6′-sialyllactose; LNT, lacto-N-tetraose; LNnT, lacto-N-neotetraose; LNFP V, lacto-N-fucopentaose-V; LNFP III, lacto-N-fucopentaose-III; LNFP II, lacto-N-fucopentaose-II; LNDFH II, lacto-N-difucohexaose II; LNnDFH II, lacto-N-neodifucohexaose II*.

There were no other statistically significant or exploratory associations at conventional level of significance of individual HMOs with LRTI (Supplementary Table 15), with the exception of 3′-SL in non-secretor milk (Supplementary Table 16) and group II milk (Supplementary Table 17) which were associated with a high risk of LRTI in the second year of life, at conventional level of statistical significance (*p* < 0.05). Overall, there were no statistically significant associations between individual HMOs and URTI in the first or second year of life (Supplementary Table 18). However, LNFPI in secretor and group I milk was associated with a decreased risk of URTI in the first and second year of life, at conventional level of significance (*p* < 0.05, Supplementary Tables 19, 20). Higher 6′-GL in non-secretor and group I milk was also associated with an increased risk of URTI in the first year of life (*p* < 0.05, Supplementary Tables 19, 20). Of note, these associations were not statistically significant following Bonferroni correction (α threshold = 0.0031).

## Discussion

The present study including 667 breastfed children investigated associations between individual HMOs measured at 6 weeks of lactation with OM, LRTI and URTI in the first or second year of life, using data from the Ulm SPATZ Health Study. Although not statistically significant, due to the large number of associations we explored, we report a tendency of lower absolute concentrations of 2′-FL and considerably higher LNT in human milk for infants with OM in the first year of life, irrespective of maternal secretor status or milk group. This difference was statistically significant and tended towards significance amongst infants who were receiving secretor milk and milk attributed to group I, respectively. However, this and all other associations were not statistically significant following adjustment for infant sex, maternal allergy, delivery mode, exclusive breastfeeding, parity, milk group and correction for multiple testing.

The absolute concentrations of 2′-FL tended to be lower while the absolute concentrations of LNT were higher in the milk of infants with OM in the second year of life. However, there were no statistically significant associations of 2′-FL with risk of OM, LRTI or URTI in the first or second year of life, in both crude and adjusted models. Similarly, protection against diarrhoea caused by *Campylobacter jejuni* was significantly associated with 2′-FL in human milk (29). That said, while the absence of functional FUT2 activity has been associated with better resistance to some pathogens such as Norovirus genotypes and Helicobacter pylori, this reportedly comes at a cost of an increased risk of infection by other pathogens affecting the respiratory, urinary or gastrointestinal systems (40). On one hand, a profile of higher LNnT and 3′-SL in the milk of HIV-infected women compared to uninfected controls has previously been reported (30, 41). On the other hand, concentrations of LNT were significantly higher in the milk of HIV-infected mothers whose HIV-exposed uninfected children survived during breastfeeding, compared to HIV-infected mothers whose HIV-exposed children died (41). Nonetheless, we attribute the inverse pattern of 2′-FL and LNT concentrations to the fact that concentrations of LNT and LNnT in human milk are core-regulated by the FUT2-dependent 2′-FL (40). In addition, although we did not find statistically significant associations, the anti-inflammatory and immunomodulatory effect of 2′-FL is well known (42–44).

Furthermore, in crude models, LNT in secretor milk was associated with a higher risk of OM in the first year of life. This association was not statistically significant following adjustment for infant sex, maternal allergy, delivery mode, exclusive breastfeeding, parity, milk group and Bonferroni correction. Likewise, another study (28) reported significantly higher levels of 2′-FL and LNT in the milk of secretor mothers whose children had symptomatic rotavirus infection. While LNFP I was higher in the milk of those who were positive for rotavirus infection compared to the rotavirus negative group of infants. Furthermore, in that same study (28), levels of 6′-SL and LNT were higher in non-secretor milk of symptomatic rotavirus group of infants, while LNFP II was significantly higher in the milk of asymptomatic rotavirus-positive group. Despite this, LNT is reportedly one of the most abundant core HMO structures present in human milk (ranging between 0.5 and 1.5 g/L in mature human milk) (19, 45). Yet, an *in vitro* study reported that LNT inhibited the growth of *Streptococcus agalactiae* (group B *Streptococcus*, GBS), a leading cause of invasive bacterial infections in newborns (46). Also, LNT has been reported to block *Entamoeba histolytica*, a major protozoan parasite in developing countries (47), from binding to epithelial cell surfaces (48). Although LNT has not yet been proposed to serve any direct antimicrobial function in infants, secretor status is useful as a stratification variable (14, 49). Thus, our results also confirm the importance of stratifying by or adjusting for maternal milk group when evaluating HMO associations with infant health outcomes.

We are only aware of two other studies that investigated similar associations with OM and respiratory problems (comprising URTI (runny nose or cold), cough, or pneumonia) (33) and acute respiratory infections (34). On one hand, Stepans et al. (33) reported associations between higher levels of LNFP II in colostrum and reduced risk of respiratory problems by 6 and 12 weeks, and non-statistically significant associations with OM. That study investigated human milk sampled at two weeks postpartum whereas we sampled human milk at 6 weeks postpartum. However, the study children (33) may have been breastfed for a longer time throughout the first year of life; while for our study the median durations of exclusive and any breastfeeding were 197 days (~28 weeks) and 122 days (~17 weeks), respectively (36). Still, it is possible that children in our study were breastfed for longer in the first year of life thus having a different baseline risk for infections compared to the other previous study. It should also be noted that only five infants were reported to have OM by 24 weeks, and the potential confounding of secretor status or milk group was not accounted for in that study (33).

On the other hand, Jorgensen et al. (34) showed a positive association between LNFP II in secretor milk with the incidence of acute respiratory infections in Malawian infants at age 6–7 months. However, this association was not statistically significant following correction for multiple testing using the Benjamin–Hochberg approach. Although they accounted for maternal secretor status, milk group status is also important to consider. Moreover, the secretor status of the infant, although difficult to measure, is also suggested to modify the association between HMOs and clinical health outcomes (14). Furthermore, the researchers in that study also assessed several other bioactive components in comparison to our study. We are only aware of one other study (50) which also investigated the relationship between HMOs and other human milk immune components, simultaneously. Firstly, while both studies (34, 50) report plausible low and moderate correlations between relative abundances of HMOs, both these studies, as well as ours, do not cover 100% of the abundance of HMOs. Of note, the relative proportions or abundance of HMOs will always differ depending on the number of individual HMOs measured and quantified despite the standardised laboratory methods human milk sample collection.

Secondly, we acknowledge that several other components, which include immunological, hormonal, enzymatic, trophic, and bioactive components in human milk offer passive protection to the growing infant (51, 52). Human milk is particularly rich in maternal cells which potentially produce cytokines and have a modulatory influence on the immunological system of new borns. Among these cellular components are macrophages and leucocytes and other immunologic molecules which are present in large amounts during early lactation, particularly in colostrum compared to mature milk (53). Most of these components in human milk can interact with each other synergistically or with other additional factors related to the mucosal or systematic immune response of the infant (9). Thus, it is plausible that other bioactive compounds as well as the multiple HMOs that were not assessed in the current study have synergistic effects to decrease or increase the risk of infection. More research is needed to determine the exact nature of the correlations that exist between HMOs and other components and their potential impact on human milk composition or volume. Granted that HMOs vary greatly within and between groups of mothers, based on secretor status or milk group, as well as time of lactation, it is plausible that any associations observed in this current study, although not statistically significant, are applicable to a specific group of infants who receive human milk for a shorter duration of lactation.

Moreover, the overall beneficial effect of HMOs in reducing other infections is widely documented (14, 20–23, 54). For instance, an observational, prospective study reported reduced diarrhoea incidence caused by *Campylobacter jejuni* and *calicivirus* in infants who received human milk containing higher levels of 2′-FL and LNDFHI, respectively (29, 55). Similarly, another study (56) reported beneficial effects of fucosyl-HMOs in reducing morbidity in Gambian infants at 4 months of age. Two observational studies on African mother-infant pairs reported reduced risk of HIV transmission (30) and decrease in mortality of HIV-exposed infants (41) receiving human milk. Specific HMO structures present in secretor milk have also been associated with direct protection against specific infections like NEC in infants (57, 58). Nonetheless, although some individual HMOs are reported to be higher in the milk of infants with some infection compared to those without infection, whether or not these are true risk factors for infection remains unknown and requires further investigation. Even so, these high concentrations may be enhanced by the effect of vaccinations against infections. For instance, 2′-FL is suggested to potentially improve the effect of vaccination against influenza virus infection (27). The clinical consequence of specific HMOs, therefore, remains to be elucidated further.

A limitation of this study is its observational nature, which makes it difficult to draw conclusions without appropriate methods for assessing causal inference when using observational data. Also, the data presented here, are naturally subject to potentially unmeasured confounding factors, which include but are not limited to secretor status of the infant. Additionally, while the novel quantification method allowed us to measure absolute HMO concentrations up to hexaose structures, it is plausible that further important oligosaccharides that have not been identified in the current study as well as in previous studies may have other important biological effects. Still, we provide a much larger sample size from a large birth cohort in comparison to the other two studies (33, 34) evaluating similar associations.

In conclusion, our results do not confirm that individual HMOs are associated with either an increased or a reduced risk of OM, LRTI and URTI in infants during early life. However, we did pick up some associations which would be statistically significant at the conventional level and may thus be subject for further, exploratory or confirmatory studies. Taken together, the evidence base is still very limited, and has several issues. More studies are needed to determine the interactions and interdependencies that exist within a broader scope of human milk components. Further studies are needed to determine whether HMOs reduce the incidence or alleviate the course of specific infections in breastfed infants. However, human studies to investigate the exact metabolic pathways and implications of single HMOs in infants are limited due to a lack of HMO availability at times, and obvious ethical issues. Hence, the need to consider or select suitable surrogate animal models in which passive maternal immune transfer is avoided. These models and research studies will allow researchers to clarify the direct effect of HMOs, in more controlled experiments and their role in infections and other health outcomes.

## Data Availability Statement

The datasets presented in this article are not readily available due to participant consent and data protection, we may not be able to share the raw data. However, the authors are open to sharing aggregate data (for instance, absolute concentrations that have been included as Supplementary Material). Requests to access the datasets should be directed to Jon.Genuneit@medizin.uni-leipzig.de.

## Ethics Statement

This study was approved by Ethics Board of Ulm University. The patients/participants provided their written informed consent to participate in this study.

## Author Contributions

LPS and JGe conceived the study question and interpreted the data and wrote the manuscript. LPS conducted the statistical analyses. MM, JGo, and BB designed the LC-MS^2^ method for HMOs analysis. JGo and BB validated the method for HMO analysis and analysed the HMOs in the lab. JGe and DR conceived and designed the Ulm SPATZ Health Study. JGe, DR, MM, and BS conceptualised the AMICA project for advanced compositional human milk analysis of SPATZ human milk samples. JGe and MM manage the AMICA project. All authors critically reviewed the manuscript and approved its final version.

## Funding

The Ulm SPATZ Health Study was funded through an unrestricted grant by the Medical Faculty of Ulm University. The current research study was funded by Danone Nutricia Research, Utrecht, The Netherlands. The funders (authors from Danone Nutricia Research) had no role in the design, analysis or writing of this article.

## Conflict of Interest

JGe is the project manager of unrestricted grants from Danone Nutricia Research to Ulm University and Leipzig University for research on other aspects of human milk composition in the Ulm birth cohort studies. MM, BS, JGo, and BB are employees of Danone Nutricia Research. However, the principal investigators JGe and DR along with LPS made final decisions on the interpretation and dissemination of results. The remaining author declares that the research was conducted in the absence of any commercial or financial relationships that could be construed as a potential conflict of interest.

## Publisher's Note

All claims expressed in this article are solely those of the authors and do not necessarily represent those of their affiliated organizations, or those of the publisher, the editors and the reviewers. Any product that may be evaluated in this article, or claim that may be made by its manufacturer, is not guaranteed or endorsed by the publisher.
