# Supplementary material for: Associations of Human Milk Oligosaccharides With Otitis Media and Lower and Upper Respiratory Tract Infections up to 2 Years: The Ulm SPATZ Health Study
Source: Front Nutr. 2021 Oct 25;8:761129. doi: 10.3389/fnut.2021.761129 (PMC8572796; doi:10.3389/fnut.2021.761129)
Supplement: Supplementary file 1 [file Table_1.docx]

Supplementary Material

**Table 1**. Absolute human milk oligosaccharide concentrations (g/L) measured at 6 weeks stratified by maternal secretor status and infant otitis media (OM) in the first year of life in the Ulm SPATZ Health Study

|  |  | Secretor milk | |  |  | Non-secretor milk | |  |
| --- | --- | --- | --- | --- | --- | --- | --- | --- |
|  |  | OM Yes (n=39) | OM No (n=352) | p |  | OM Yes (n=11) | OM No (n=88) | p |
| Lactose | |  |  |  |  |  |  |  |
|  | Mean (SD) | 66.7 (3.93) | 66.4 (3.74) |  |  | 67.1 (5.70) | 67.3 (3.42) |  |
|  | Median [min, max] | 67.0 [58.0, 73.0] | 67.0 [52.0, 77.0] | 0.701 |  | 69.0 [58.0, 74.0] | 68.0 [56.0, 74.0] | 0.654 |
| 2'-FL | |  |  |  |  |  |  |  |
|  | Mean (SD) | 2.44 (1.02) | 2.86 (0.10) |  |  | 0.13 (0) | 0.13 (0) |  |
|  | Median [min, max] | 2.40 [0.36, 6.30] | 2.70 [0.13, 6.60] | 0.009 |  | 0.13 [0.13, 0.13] | 0.13 [0.13, 0.13] |  |
| 3-FL | |  |  |  |  |  |  |  |
|  | Mean (SD) | 0.48 (0.28) | 0.45 (0.27) |  |  | 1.55 (0.46) | 1.52 (0.53) |  |
|  | Median [min, max] | 0.38 [0.11, 1.10] | 0.41 [0.03, 1.90] | 0.88 |  | 1.70 [0.63, 2.10] | 1.60 [0.08, 2.90] | 0.797 |
| 3'-SL | |  |  |  |  |  |  |  |
|  | Mean (SD) | 0.15 (0.04) | 0.15 (0.04) |  |  | 0.18 (0.05) | 0.18 (0.05) |  |
|  | Median [min, max] | 0.14 [0.08, 0.27] | 0.14 [0.05, 0.26] | 0.681 |  | 0.16 [0.11, 0.27] | 0.17 [0.07, 0.32] | 1 |
| 6'-GL | |  |  |  |  |  |  |  |
|  | Mean (SD) | 0.02 (0.01) | 0.02 (0.01) |  |  | 0.03 (0.04) | 0.02 (0.01) |  |
|  | Median [min, max] | 0.01 [0.01, 0.05] | 0.02 [0.004, 0.09] | 0.567 |  | 0.02 [0.01, 0.15] | 0.02 [0.01, 0.04] | 0.049 |
| DFL | |  |  |  |  |  |  |  |
|  | Mean (SD) | 0.20 (0.14) | 0.23 (0.17) |  |  | 0.01 (0) | 0.01 (0) |  |
|  | Median [min, max] | 0.16 [0.06, 0.72] | 0.20 [0.01, 1.80] | 0.055 |  | 0.01 [0.01, 0.01] | 0.01 [0.01, 0.01] |  |
| 6'-SL | |  |  |  |  |  |  |  |
|  | Mean (SD) | 0.26 (0.10) | 0.26 (0.11) |  |  | 0.24 (0.09) | 0.25 (0.101) |  |
|  | Median [min, max] | 0.24 [0.07, 0.54] | 0.24 [0.06, 0.73] | 0.986 |  | 0.23 [0.11, 0.39] | 0.24 [0.07, 0.55] | 0.648 |
| LNT | |  |  |  |  |  |  |  |
|  | Mean (SD) | 1.04 (0.50) | 0.81 (0.39) |  |  | 1.29 (0.48) | 1.35 (0.61) |  |
|  | Median [min, max] | 0.98 [0.15, 2.60] | 0.76 [0.14, 2.90] | 0.0019 |  | 1.10 [0.77, 2.10] | 1.30 [0.24, 3.10] | 0.88 |
| LNnT | |  |  |  |  |  |  |  |
|  | Mean (SD) | 0.09 (0.05) | 0.10 (0.06) |  |  | 0.03 (0.02) | 0.03 (0.02) |  |
|  | Median [min, max] | 0.08 [0.03, 0.26] | 0.09 [0.01, 0.34] | 0.467 |  | 0.02 [0.01, 0.06] | 0.02 [0.01, 0.13] | 0.497 |
| LNFP I | |  |  |  |  |  |  |  |
|  | Mean (SD) | 0.63 (0.47) | 0.60 (0.44) |  |  | 0.04 (0) | 0.04 (0) |  |
|  | Median [min, max] | 0.66 [0.04, 2.20] | 0.50 [0.04, 3.00] | 0.737 |  | 0.04 [0.04, 0.04] | 0.04 [0.04, 0.04] |  |
| LNFP V | |  |  |  |  |  |  |  |
|  | Mean (SD) | 0.03 (0.02) | 0.02 (0.01) |  |  | 0.11 (0.03) | 0.10 (0.04) |  |
|  | Median [min, max] | 0.02 [0.01, 0.08] | 0.02 [0.01, 0.09] | 0.082 |  | 0.10 [0.07, 0.17] | 0.09 [0.01, 0.24] | 0.227 |
| LNFP III | |  |  |  |  |  |  |  |
|  | Mean (SD) | 0.18 (0.06) | 0.17 (0.07) |  |  | 0.24 (0.08) | 0.21 (0.07) |  |
|  | Median [min, max] | 0.18 [0.06, 0.34] | 0.17 [0.04, 0.48] | 0.677 |  | 0.23 [0.15, 0.43] | 0.20 [0.08, 0.43] | 0.147 |
| LNFP II | |  |  |  |  |  |  |  |
|  | Mean (SD) | 0.23 (0.20) | 0.18 (0.15) |  |  | 1.07 (0.32) | 0.97 (0.46) |  |
|  | Median [min, max] | 0.14 [0.04, 0.93] | 0.14 [0.04, 1.20] | 0.372 |  | 1.10 [0.58, 1.60] | 0.94 [0.04, 2.50] | 0.376 |
| LNDFH I | |  |  |  |  |  |  |  |
|  | Mean (SD) | 0.64 (0.30) | 0.66 (0.33) |  |  | 0.02 (0) | 0.02 (0) |  |
|  | Median [min, max] | 0.65 [0.02, 1.50] | 0.63 [0.02, 1.90] | 0.574 |  | 0.02 [0.02, 0.02] | 0.02 [0.02, 0.02] |  |
| LNDFH II + LNnDFH II | |  |  |  |  |  |  |  |
|  | Mean (SD) | 0.02 (0.02) | 0.02 (0.02) |  |  | 0.27 (0.14) | 0.24 (0.14) |  |
|  | Median [min, max] | 0.01 [0.01, 0.11] | 0.01 [0.01, 0.18] | 0.487 |  | 0.32 [0.07, 0.46] | 0.23 [0.01, 0.73] | 0.341 |
| Sum of HMOs | |  |  |  |  |  |  |  |
|  | Mean (SD) | 6.38 (1.27) | 6.52 (1.15) |  |  | 5.21 (0.93) | 5.05 (1.13) |  |
|  | Median [min, max] | 6.44 [3.78, 9.69] | 6.41 [3.58, 11.6] | 0.546 |  | 5.48 [3.70, 6.80] | 4.98 [2.41, 8.40] | 0.593 |

P values derived from Wilcoxon sum-rank test comparing HMO concentrations between infants with and without otitis media at 1 year within the secretor and non-secretor milk groups. Bonferroni-adjusted level of statistical significance is α = 0.05/16 = 0.0031. OM- Otitis media; HMO, human milk oligosaccharides. 2’-FL, 2’-fucosyllactose; 3-FL, 3-fucosyllactose; 3’-SL, 3’-sialyllactose; 6'-GL, 6’-Galactosyllactose; DFL, 3,2’-difucosyllactose; 6’-SL; 6’-sialyllactose; LNT, lacto-N-tetraose; LNnT, lacto-N-neotetraose; LNFP I, lacto-N-fucopentaose-I; LNFP V, lacto-N-fucopentaose-V; LNFP III, lacto-N-fucopentaose-III; LNFP II, lacto-N-fucopentaose-II; LNDFH I, lacto-N-difucohexaose I; LNDFH II, lacto-N-difucohexaose II; LNnDFH II, lacto-N-neodifucohexaose II.

**Table 2**: Absolute human milk oligosaccharide concentrations (g/L) measured at 6 weeks stratified by maternal secretor status and infant otitis media (OM) in the second year of life in the Ulm SPATZ Health Study

|  |  | Secretor milk | |  | Non-secretor milk | |  |
| --- | --- | --- | --- | --- | --- | --- | --- |
|  |  | OM Yes(n=113) | OM No (n=287) | p | OM Yes (n=27) | OM No (n=75) | p |
| Lactose | |  |  |  |  |  |  |
|  | Mean (SD) | 66.3 (3.90) | 66.6 (3.75) |  | 67.5 (4.34) | 67.1 (3.39) |  |
|  | Median [min, max] | 67.0 [56.0, 77.0] | 67.0 [52.0, 74.0] | 0.23 | 69.0 [58.0, 74.0] | 67.0 [56.0, 74.0] | 0.30 |
| 2'-FL | |  |  |  |  |  |  |
|  | Mean (SD) | 2.77 (0.94) | 2.85 (1.03) |  | 0.13 (0) | 0.13 (0) |  |
|  | Median [min, max] | 2.70 [0.36, 6.30] | 2.70 [0.13, 6.60] | 0.67 | 0.13 [0.13, 0.13] | 0.13 [0.13, 0.13] |  |
| 3-FL | |  |  |  |  |  |  |
|  | Mean (SD) | 0.44 (0.26) | 0.46 (0.27) |  | 1.37 (0.63) | 1.56 (0.48) |  |
|  | Median [min, max] | 0.38 [0.03, 1.20] | 0.41 [0.03, 1.90] | 0.31 | 1.40 [0.08, 2.90] | 1.60 [0.15, 2.60] | 0.14 |
| 3'-SL | |  |  |  |  |  |  |
|  | Mean (SD) | 0.15 (0.04) | 0.15 (0.04) |  | 0.18 (0.06) | 0.18 (0.05) |  |
|  | Median [min, max] | 0.14 [0.05, 0.27] | 0.14 [0.07, 0.26] | 0.81 | 0.17 [0.07, 0.30] | 0.17 [0.09, 0.32] | 0.78 |
| 6'-GL | |  |  |  |  |  |  |
|  | Mean (SD) | 0.02 (0.01) | 0.02 (0.01) |  | 0.03 (0.03) | 0.02 (0.01) |  |
|  | Median [min, max] | 0.02 [0.01, 0.05] | 0.02 [0.004, 0.09] | 0.47 | 0.02 [0.01, 0.15] | 0.02 [0.01, 0.04] | 0.0037 |
| DFL | |  |  |  |  |  |  |
|  | Mean (SD) | 0.21 (0.13) | 0.23 (0.17) |  | 0.01 (0) | 0.01 (0) |  |
|  | Median [min, max] | 0.18 [0.01, 0.72] | 0.20 [0.02, 1.80] | 0.23 | 0.01 [0.01, 0.01] | 0.01 [0.01, 0.01] |  |
| 6'-SL | |  |  |  |  |  |  |
|  | Mean (SD) | 0.25 (0.10) | 0.27 (0.12) |  | 0.24 (0.09) | 0.25 (0.10) |  |
|  | Median [min, max] | 0.23 [0.07, 0.54] | 0.24 [0.06, 0.73] | 0.45 | 0.24 [0.07, 0.42] | 0.23 [0.08, 0.55] | 0.51 |
| LNT | |  |  |  |  |  |  |
|  | Mean (SD) | 0.88 (0.44) | 0.817 (0.40) |  | 1.41 (0.65) | 1.32 (0.57) |  |
|  | Median [min, max] | 0.84 [0.15, 2.60] | 0.76 [0.14, 2.90] | 0.24 | 1.30 [0.24, 3.10] | 1.20 [0.36, 2.90] | 0.57 |
| LNnT | |  |  |  |  |  |  |
|  | Mean (SD) | 0.10 (0.06) | 0.10 (0.06) |  | 0.03 (0.03) | 0.02 (0.02) |  |
|  | Median [min, max] | 0.08 [0.01, 0.34] | 0.06 [0.01, 0.33] | 0.94 | 0.02 [0.01, 0.10] | 0.02 [0.01, 0.13] | 0.37 |
| LNFP I | |  |  |  |  |  |  |
|  | Mean (SD) | 0.65 (0.47) | 0.60 (0.45) |  | 0.04 (0) | 0.04 (0) |  |
|  | Median [min, max] | 0.59 [0.04, 2.70] | 0.47 [0.04, 3.00] | 0.31 | 0.04 [0.04, 0.04] | 0.04 [0.04, 0.04] |  |
| LNFP V | |  |  |  |  |  |  |
|  | Mean (SD) | 0.02 (0.02) | 0.02 (0.01) |  | 0.10 (0.04) | 0.10 (0.04) |  |
|  | Median [min, max] | 0.02 [0.01, 0.08] | 0.02 [0.01, 0.09] | 0.7 | 0.09 [0.01, 0.17] | 0.09 [0.02, 0.24] | 0.50 |
| LNFP III | |  |  |  |  |  |  |
|  | Mean (SD) | 0.17 (0.07) | 0.18 (0.07) |  | 0.22 (0.08) | 0.21 (0.07) |  |
|  | Median [min, max] | 0.16 [0.05, 0.39] | 0.17 [0.04, 0.48] | 0.27 | 0.19 [0.11, 0.43] | 0.20 [0.08, 0.43] | 0.48 |
| LNFP II | |  |  |  |  |  |  |
|  | Mean (SD) | 0.19 (0.16) | 0.19 (0.15) |  | 0.90 (0.43) | 0.10 (0.46) |  |
|  | Median [min, max] | 0.13 [0.04, 0.93] | 0.14 [0.04, 1.20] | 0.49 | 0.99 [0.04, 1.60] | 0.93 [0.04, 2.50] | 0.65 |
| LNDFH I | |  |  |  |  |  |  |
|  | Mean (SD) | 0.67 (0.32) | 0.66 (0.33) |  | 0.02 (0) | 0.02 (0) |  |
|  | Median [min, max] | 0.65 [0.02, 1.70] | 0.63 [0.02, 1.90] | 0.88 | 0.02 [0.02, 0.02] | 0.02 [0.02, 0.02] |  |
| LNDFH II + LNnDFH II | |  |  |  |  |  |  |
|  | Mean (SD) | 0.02 (0.02) | 0.02 (0.02) |  | 0.22 (0.13) | 0.25 (0.14) |  |
|  | Median [min, max] | 0.01 [0.01, 0.11] | 0.01 [0.01, 0.18] | 0.62 | 0.18 [0.01, 0.46] | 0.23 [0.01, 0.73] | 0.49 |
| Sum of HMOs | |  |  |  |  |  |  |
|  | Mean (SD) | 6.51 (1.17) | 6.54 (1.16) |  | 4.89 (1.13) | 5.09 (1.11) |  |
|  | Median [min, max] | 6.43 [3.78, 9.80] | 6.44 [3.58, 11.6] | 0.83 | 4.68 [2.41, 6.80] | 4.97 [2.76, 8.40] | 0.62 |

P values derived from Wilcoxon sum-rank test comparing HMO concentrations between infants with and without otitis media at 2 years between the secretor and non-secretor milk groups Bonferroni-adjusted level of statistical significance is α = 0.05/16 = 0.0031. OM- Otitis media; HMO, human milk oligosaccharides. 2’-FL, 2’-fucosyllactose; 3-FL, 3-fucosyllactose; 3’-SL, 3’-sialyllactose; 6'-GL, 6’-Galactosyllactose; DFL, 3,2’-difucosyllactose; 6’-SL; 6’-sialyllactose; LNT, lacto-N-tetraose; LNnT, lacto-N-neotetraose; LNFP I, lacto-N-fucopentaose-I; LNFP V, lacto-N-fucopentaose-V; LNFP III, lacto-N-fucopentaose-III; LNFP II, lacto-N-fucopentaose-II; LNDFH I, lacto-N-difucohexaose I; LNDFH II, lacto-N-difucohexaose II; LNnDFH II, lacto-N-neodifucohexaose II

**Table 3**: Absolute human milk oligosaccharide concentrations (g/L) measured at 6 weeks stratified by maternal milk group and infant otitis media (OM) in the first year of life in the Ulm SPATZ Health Study

|  |  | Group I milk | | p | Group II milk | | p |
| --- | --- | --- | --- | --- | --- | --- | --- |
|  |  | OM Yes (n=37) | OM No (320) |  | OM Yes (n=11) | OM No (n=84) |  |
| Lactose | |  |  |  |  |  |  |
|  | Mean (SD) | 66.7 (4.03) | 66.5 (3.74) |  | 67.1 (5.70) | 67.2 (3.48) |  |
|  | Median [min, max] | 67.0 [58.0, 73.0] | 67.0 [52.0, 77.0] | 0.69 | 69.0 [58.0, 74.0] | 68.0 [56.0, 74.0] | 0.65 |
| 2'-FL | |  |  |  |  |  |  |
|  | Mean (SD) | 2.42 (1.02) | 2.72 (0.88) |  | 0.13 (0) | 0.13 (0) |  |
|  | Median [min, max] | 2.40 [0.36, 6.30] | 2.65 [0.13, 5.70] | 0.03 | 0.13 [0.13, 0.13] | 0.13 [0.13, 0.13] |  |
| 3-FL | |  |  |  |  |  |  |
|  | Mean (SD) | 0.50 (0.28) | 0.49 (0.25) |  | 1.55 (0.46) | 1.58 (0.46) |  |
|  | Median [min, max] | 0.39 [0.14, 1.10] | 0.43 [0.09, 1.90] | 0.77 | 1.70 [0.63, 2.10] | 1.60 [0.76, 2.90] | 0.99 |
| 3'-SL | |  |  |  |  |  |  |
|  | Mean (SD) | 0.15 (0.04) | 0.15 (0.04) |  | 0.18 (0.05) | 0.18 (0.05) |  |
|  | Median [min, max] | 0.14 [0.08, 0.27] | 0.14 [0.05, 0.26] | 0.78 | 0.16 [0.11, 0.27] | 0.17 [0.07, 0.3] | 0.96 |
| 6'-GL | |  |  |  |  |  |  |
|  | Mean (SD) | 0.02 (0.01) | 0.02 (0.01) |  | 0.03 (0.04) | 0.02 (0.01) |  |
|  | Median [min, max] | 0.01 [0.01, 0.05] | 0.02 [0.004, 0.09] | 0.32 | 0.02 [0.01, 0.15] | 0.02 [0.01, 0.04] | 0.04 |
| DFL | |  |  |  |  |  |  |
|  | Mean (SD) | 0.21 (0.14) | 0.24 (0.16) |  | 0.01 (0) | 0.01 (0) |  |
|  | Median [min, max] | 0.16 [0.06, 0.72] | 0.21 [0.05, 1.80] | 0.01 | 0.01 [0.01, 0.01] | 0.01 [0.01, 0.01] |  |
| 6'-SL | |  |  |  |  |  |  |
|  | Mean (SD) | 0.26 (0.10) | 0.26 (0.10) |  | 0.24 (0.09) | 0.25 (0.10) |  |
|  | Median [min, max] | 0.24 [0.07, 0.54] | 0.24 [0.06, 0.63] | 0.99 | 0.23 [0.11, 0.39] | 0.23 [0.07, 0.55] | 0.69 |
| LNT | |  |  |  |  |  |  |
|  | Mean (SD) | 1.03 (0.49) | 0.83 (0.39) |  | 1.29 (0.48) | 1.32 (0.60) |  |
|  | Median [min, max] | 0.98 [0.15, 2.60] | 0.77 [0.14, 2.90] | 0.0067 | 1.10 [0.77, 2.10] | 1.20 [0.24, 3.10] | 0.98 |
| LNnT | |  |  |  |  |  |  |
|  | Mean (SD) | 0.09 (0.05) | 0.10 (0.06) |  | 0.03 (0.02) | 0.03 (0.02) |  |
|  | Median [min, max] | 0.08 [0.03, 0.26] | 0.09 [0.01, 0.34] | 0.37 | 0.02 [0.01, 0.06] | 0.02 [0.01, 0.13] | 0.41 |
| LNFP I | |  |  |  |  |  |  |
|  | Mean (SD) | 0.58 (0.40) | 0.54 (0.37) |  | 0.04 (0) | 0.04 (0) |  |
|  | Median [min, max] | 0.59 [0.04, 1.40] | 0.44 [0.04, 1.90] | 0.57 | 0.04 [0.04 0.04] | 0.04 [0.04, 0.04] |  |
| LNFP V | |  |  |  |  |  |  |
|  | Mean (SD) | 0.03 (0.02) | 0.02 (0.01) |  | 0.11 (0.03) | 0.10 (0.04) |  |
|  | Median [min, max] | 0.02 [0.01, 0.08] | 0.02 [0.01, 0.09] | 0.16 | 0.10 [0.07, 0.17] | 0.09 [0.03, 0.24] | 0.25 |
| LNFP III | |  |  |  |  |  |  |
|  | Mean (SD) | 0.17 (0.06) | 0.18 (0.07) |  | 0.24 (0.08) | 0.20 (0.07) |  |
|  | Median [min, max] | 0.16 [0.06, 0.34] | 0.18 [0.04, 0.48] | 0.58 | 0.23 [0.15, 0.43] | 0.20 [0.08, 0.43] | 0.12 |
| LNFP II | |  |  |  |  |  |  |
|  | Mean (SD) | 0.24 (0.20) | 0.20 (0.15) |  | 1.07 (0.32) | 1.02 (0.42) |  |
|  | Median [min, max] | 0.15 [0.04, 0.93] | 0.15 [0.04, 1.20] | 0.55 | 1.10 [0.58, 1.60] | 0.96 [0.24, 2.50] | 0.50 |
| LNDFH I | |  |  |  |  |  |  |
|  | Mean (SD) | 0.67 (0.27) | 0.72 (0.23) |  | 0.02 (0) | 0.02 (0) |  |
|  | Median [min, max] | 0.65 [0.19, 1.50] | 0.67 [0.07, 1.90] | 0.29 | 0.02 [0.02, 0.02] | 0.02 [0.02, 0.02] |  |
| LNDFH II + LNnDFH II | |  |  |  |  |  |  |
|  | Mean (SD) | 0.02 (0.02) | 0.02 (0.02) |  | 0.27 (0.14) | 0.25 (0.13) |  |
|  | Median [min, max] | 0.01 [0.01, 0.11] | 0.01 [0.01, 0.18] | 0.60 | 0.32 [0.07, 0.46] | 0.23 [0.04, 0.73] | 0.46 |
| Sum of HMOs | |  |  |  |  |  |  |
|  | Mean (SD) | 6.37 (1.21) | 6.48 (1.11) |  | 5.21 (0.93) | 5.14 (1.08) |  |
|  | Median [min, max] | 6.44 [3.78, 9.69] | 6.38 [3.58, 11.1] | 0.66 | 5.48 [3.70, 6.80] | 5.01 [2.76, 8.40] | 0.75 |

P values derived from Wilcoxon sum-rank test comparing HMO concentrations between infants with and without otitis media at 1 year within group I and group II milk. Bonferroni-adjusted level of statistical significance is α = 0.05/16 = 0.0031. OM- Otitis media; HMO, human milk oligosaccharides. 2’-FL, 2’-fucosyllactose; 3-FL, 3-fucosyllactose; 3’-SL, 3’-sialyllactose; 6'-GL, 6’-Galactosyllactose; DFL, 3,2’-difucosyllactose; 6’-SL; 6’-sialyllactose; LNT, lacto-N-tetraose; LNnT, lacto-N-neotetraose; LNFP I, lacto-N-fucopentaose-I; LNFP V, lacto-N-fucopentaose-V; LNFP III, lacto-N-fucopentaose-III; LNFP II, lacto-N-fucopentaose-II; LNDFH I, lacto-N-difucohexaose I; LNDFH II, lacto-N-difucohexaose II; LNnDFH II, lacto-N-neodifucohexaose II

**Table 4**: Absolute human milk oligosaccharide concentrations (g/L) measured at 6 weeks stratified by maternal milk group and infant otitis media (OM) in the second year of life in the Ulm SPATZ Health Study

|  |  | Group I milks | | p | Group II milks | | p |
| --- | --- | --- | --- | --- | --- | --- | --- |
|  |  | OM Yes (n=105) | OM No (n=262) |  | OM Yes (n=24) | OM No (n=73) |  |
| Lactose | |  |  |  |  |  |  |
|  | Mean (SD) | 66.3 (3.88) | 66.6 (3.74) |  | 67.3 (4.57) | 67.2 (3.44) |  |
|  | Median [min, max] | 66.0 [56.0, 77.0] | 67.0 [52.0, 74.0] | 0.21 | 68.5 [58.0, 74.0] | 67.0 [56.0, 74.0] | 0.50 |
| 2'-FL | |  |  |  |  |  |  |
|  | Mean (SD) | 2.68 (0.86) | 2.71 (0.91) |  | 0.13 (0) | 0.13 (0) |  |
|  | Median [min, max] | 2.70 [0.36, 6.30] | 2.60 [0.13, 5.70] | 0.98 | 0.13 [0.13, 0.13] | 0.13 [0.13, 0.13] |  |
| 3-FL | |  |  |  |  |  |  |
|  | Mean (SD) | 0.46 (0.25) | 0.49 (0.25) |  | 1.51 (0.49) | 1.60 (0.44) |  |
|  | Median [min, max] | 0.40 [0.09, 1.20] | 0.44 [0.10, 1.90] | 0.16 | 1.45 [0.63, 2.90] | 1.60 [0.78, 2.60] | 0.37 |
| 3'-SL | |  |  |  |  |  |  |
|  | Mean (SD) | 0.15 (0.04) | 0.15 (0.04) |  | 0.18 (0.06) | 0.18 (0.05) |  |
|  | Median [min, max] | 0.14 [0.05, 0.27] | 0.14 [0.07, 0.26] | 0.82 | 0.17 [0.07, 0.30] | 0.17 [0.09, 0.32] | 0.67 |
| 6'-GL | |  |  |  |  |  |  |
|  | Mean (SD) | 0.02 (0.01) | 0.02 (0.01) |  | 0.03 (0.03) | 0.02 (0.01) |  |
|  | Median [min, max] | 0.01 [0.01, 0.05] | 0.02 [0.004, 0.09] | 0.38 | 0.02 [0.01, 0.15] | 0.02 [0.01, 0.04] | 0.02 |
| DFL | |  |  |  |  |  |  |
|  | Mean (SD) | 0.22 (0.13) | 0.24 (0.17) |  | 0.01 (0) | 0.01 (0) |  |
|  | Median [min, max] | 0.19 [0.06, 0.72] | 0.21 [0.05, 1.80] | 0.13 | 0.01 [0.01, 0.01] | 0.01 [0.01, 0.01] |  |
| 6'-SL | |  |  |  |  |  |  |
|  | Mean (SD) | 0.25 (0.10) | 0.26 (0.10) |  | 0.23 (0.09) | 0.25 (0.10) |  |
|  | Median [min, max] | 0.23 [0.07, 0.54] | 0.24 [0.06, 0.63] | 0.61 | 0.21 [0.07, 0.42] | 0.23 [0.08, 0.55] | 0.26 |
| LNT | |  |  |  |  |  |  |
|  | Mean (SD) | 0.89 (0.44) | 0.83 (0.39) |  | 1.37 (0.67) | 1.30 (0.56) |  |
|  | Median [min, max] | 0.85 [0.15, 2.60] | 0.78 [0.14, 2.90] | 0.28 | 1.30 [0.24, 3.10] | 1.20 [0.36, 2.90] | 0.73 |
| LNnT | |  |  |  |  |  |  |
|  | Mean (SD) | 0.10 (0.06) | 0.10 (0.06) |  | 0.03 (0.02) | 0.02 (0.02) |  |
|  | Median [min, max] | 0.08 [0.01, 0.34] | 0.09 [0.01, 0.33] | 0.92 | 0.02 [0.01, 0.10] | 0.02 [0.01, 0.13] | 0.67 |
| LNFP I | |  |  |  |  |  |  |
|  | Mean (SD) | 0.61 (0.41) | 0.53 (0.36) |  | 0.04 (0) | 0.04 (0) |  |
|  | Median [min, max] | 0.57 [0.04, 1.70] | 0.44 [0.04, 1.90] | 0.13 | 0.04 [0.04, 0.04] | 0.04 [0.04, 0.04] |  |
| LNFP V | |  |  |  |  |  |  |
|  | Mean (SD) | 0.02 (0.02) | 0.02 (0.01) |  | 0.10 (0.04) | 0.09 (0.04) |  |
|  | Median [min, max] | 0.02 [0.01, 0.08] | 0.02 [0.01, 0.09] | 0.56 | 0.10 [0.04, 0.17] | 0.09 [0.03, 0.24] | 0.15 |
| LNFP III | |  |  |  |  |  |  |
|  | Mean (SD) | 0.17 (0.07) | 0.18 (0.07) |  | 0.23 (0.07) | 0.20 (0.07) |  |
|  | Median [min, max] | 0.16 [0.05, 0.39] | 0.18 [0.04, 0.48] | 0.19 | 0.20 [0.13, 0.43] | 0.20 [0.08, 0.43] | 0.27 |
| LNFP II | |  |  |  |  |  |  |
|  | Mean (SD) | 0.20 (0.16) | 0.20 (0.15) |  | 1.01 (0.321) | 1.02 (0.43) |  |
|  | Median [min, max] | 0.13 [0.04, 0.93] | 0.150 [0.04, 1.20] | 0.33 | 1.00 [0.44, 1.60] | 0.95 [0.24, 2.50] | 0.83 |
| LNDFH I | |  |  |  |  |  |  |
|  | Mean (SD) | 0.71 (0.28) | 0.72 (0.27) |  | 0.02 (0) | 0.02 (0) |  |
|  | Median [min, max] | 0.67 [0.19, 1.70] | 0.66 [0.07, 1.90] | 0.64 | 0.02 [0.02, 0.02] | 0.02 [0.02, 0.02] |  |
| LNDFH II + LNnDFH II | |  |  |  |  |  |  |
|  | Mean (SD) | 0.02 (0.02) | 0.02 (0.02) |  | 0.24 (0.12) | 0.25 (0.14) |  |
|  | Median [min, max] | 0.01 [0.01, 0.11] | 0.01 [0.01, 0.18] | 0.55 | 0.22 [0.07, 0.46] | 0.23 [0.04, 0.73] | 0.98 |
| Sum of HMOs | |  |  |  |  |  |  |
|  | Mean (SD) | 6.49 (1.10) | 6.47 (1.12) |  | 5.13 (0.93) | 5.13 (1.09) |  |
|  | Median [min, max] | 6.43 [3.78, 9.69] | 6.37 [3.58, 11.1] | 0.82 | 5.33 [3.43, 6.80] | 4.98 [2.76, 8.40] | 0.88 |

P values derived from Wilcoxon sum-rank test comparing HMO concentrations between infants with and without otitis media at 2 years within group I and group II milk. Bonferroni-adjusted level of statistical significance is α = 0.05/16 = 0.0031. OM- Otitis media; HMO, human milk oligosaccharides. 2’-FL, 2’-fucosyllactose; 3-FL, 3-fucosyllactose; 3’-SL, 3’-sialyllactose; 6'-GL, 6’-Galactosyllactose; DFL, 3,2’-difucosyllactose; 6’-SL; 6’-sialyllactose; LNT, lacto-N-tetraose; LNnT, lacto-N-neotetraose; LNFP I, lacto-N-fucopentaose-I; LNFP V, lacto-N-fucopentaose-V; LNFP III, lacto-N-fucopentaose-III; LNFP II, lacto-N-fucopentaose-II; LNDFH I, lacto-N-difucohexaose I; LNDFH II, lacto-N-difucohexaose II; LNnDFH II, lacto-N-neodifucohexaose II

**Table 5**. Absolute human milk oligosaccharide concentrations (g/L) measured at 6 weeks and lower respiratory tract infections (LRTI) in infants in the first or second year of life in the Ulm SPATZ Health Study

|  |  | LRTI in the first year of life | |  |  | LRTI in the second year of life | |  | |
| --- | --- | --- | --- | --- | --- | --- | --- | --- | --- |
|  |  | LRTI Yes (n=146) | LRTI No (n=346) | p |  | LRTI Yes (n=240) | LRTI No (n=298) | | p |
| Lactose | |  |  |  |  |  |  |  | |
|  | Mean (SD) | 66.4 (4.19) | 66.7 (3.57) |  |  | 66.6 (3.86) | 66.7 (3.62) | 0.659 | |
|  | Median [min, max] | 67.0 [53.0, 74.0] | 67.0 [52.0, 77.0] | 0.84 |  | 67.0 [53.0, 74.0] | 67.0 [52.0, 77.0] |  | |
| 2'-FL | |  |  |  |  |  |  |  | |
|  | Mean (SD) | 2.15 (1.45) | 2.33 (1.38) |  |  | 2.20 (1.42) | 2.38 (1.36) | 0.065 | |
|  | Median [min, max] | 2.30 [0.13, 6.60] | 2.50 [0.13, 6.00] | 0.12 |  | 2.40 [0.13, 6.60] | 2.50 [0.13, 5.90] |  | |
| 3-FL | |  |  |  |  |  |  |  | |
|  | Mean (SD) | 0.69 (0.54) | 0.66 (0.54) |  |  | 0.69 (0.57) | 0.63 (0.50) | 0.334 | |
|  | Median [min, max] | 0.52 [0.04, 2.60] | 0.49 [0.03, 2.90] | 0.40 |  | 0.51 [0.03, 2.90] | 0.47 [0.03, 2.40] |  | |
| 3'-SL | |  |  |  |  |  |  |  | |
|  | Mean (SD) | 0.15 (0.04) | 0.15 (0.04) |  |  | 0.15 (0.04) | 0.15 (0.04) | 0.47 | |
|  | Median [min, max] | 0.15 [0.08, 0.29] | 0.15 [0.05, 0.32] | 0.39 |  | 0.15 [0.05, 0.30] | 0.15 [0.07, 0.32] |  | |
| 6'-GL | |  |  |  |  |  |  |  | |
|  | Mean (SD) | 0.02 (0.01) | 0.02 (0.01) |  |  | 0.02 (0.01) | 0.02 (0.01) | 0.845 | |
|  | Median [min, max] | 0.02 [0.01, 0.05] | 0.02 [0.004, 0.15] | 0.59 |  | 0.02 [0.004, 0.05] | 0.02 [0.01, 0.15] |  | |
| DFL | |  |  |  |  |  |  |  | |
|  | Mean (SD) | 0.19 (0.18) | 0.18 (0.17) |  |  | 0.17 (0.16) | 0.19 (0.17) | 0.367 | |
|  | Median [min, max] | 0.17 [0.01, 1.40] | 0.17 [0.01, 1.80] | 0.91 |  | 0.17 [0.01, 1.40] | 0.17 [0.01, 1.80] |  | |
| 6'-SL | |  |  |  |  |  |  |  | |
|  | Mean (SD) | 0.26 (0.11) | 0.26 (0.10) |  |  | 0.26 (0.11) | 0.26 (0.10) | 0.901 | |
|  | Median [min, max] | 0.24 [0.07, 0.63] | 0.24 [0.06, 0.63] | 0.83 |  | 0.24 [0.07, 0.73] | 0.24 [0.05, 0.63] |  | |
| LNT | |  |  |  |  |  |  |  | |
|  | Mean (SD) | 0.99 (0.56) | 0.93 (0.47) |  |  | 0.95 (0.51) | 0.92 (0.47) | 0.56 | |
|  | Median [min, max] | 0.87 [0.15, 3.10] | 0.84 [0.14, 2.90] | 0.55 |  | 0.87 [0.15, 3.10] | 0.82 [0.14, 2.90] |  | |
| LNnT | |  |  |  |  |  |  |  | |
|  | Mean (SD) | 0.08 (0.06) | 0.09 (0.06) |  |  | 0.08 (0.06) | 0.09 (0.06) | 0.286 | |
|  | Median [min, max] | 0.07 [0.01, 0.30] | 0.07 [0.01, 0.35] | 0.28 |  | 0.07 [0.01, 0.35] | 0.07 [0.01, 0.33] |  | |
| LNFP I | |  |  |  |  |  |  |  | |
|  | Mean (SD) | 0.47 (0.49) | 0.50 (0.44) |  |  | 0.48 (0.46) | 0.51 (0.46) | 0.259 | |
|  | Median [min, max] | 0.33 [0.04, 3.00] | 0.41 [0.04, 2.40] | 0.25 |  | 0.35 [0.04, 3.00] | 0.41 [0.04, 2.70] |  | |
| LNFP V | |  |  |  |  |  |  |  | |
|  | Mean (SD) | 0.04 (0.04) | 0.04 (0.04) |  |  | 0.04 (0.04) | 0.03 (0.04) | 0.23 | |
|  | Median [min, max] | 0.02 [0.01, 0.17] | 0.02 [0.01, 0.24] | 0.19 |  | 0.02 [0.01, 0.17] | 0.02 [0.01, 0.24] |  | |
| LNFP III | |  |  |  |  |  |  |  | |
|  | Mean (SD) | 0.18 (0.08) | 0.18 (0.07) |  |  | 0.18 (0.07) | 0.18 (0.07) | 0.285 | |
|  | Median [min, max] | 0.18 [0.05, 0.43] | 0.18 [0.04, 0.48] | 0.85 |  | 0.17 [0.05, 0.43] | 0.18 [0.04, 0.48] |  | |
| LNFP II | |  |  |  |  |  |  |  | |
|  | Mean (SD) | 0.37 (0.39) | 0.34 (0.40) |  |  | 0.36 (0.39) | 0.32 (0.39) | 0.163 | |
|  | Median [min, max] | 0.19 [0.04, 2.00] | 0.17 [0.04, 2.50] | 0.26 |  | 0.19 [0.04, 2.00] | 0.160 [0.04, 2.50] | | |
| LNDFH I | |  |  |  |  |  |  |  | |
|  | Mean (SD) | 0.53 (0.40) | 0.54 (0.38) |  |  | 0.53 (0.39) | 0.54 (0.37) | 0.888 | |
|  | Median [min, max] | 0.59 [0.02, 1.70] | 0.57 [0.02, 1.90] | 0.93 |  | 0.58 [0.02, 1.70] | 0.57 [0.02, 1.90] |  | |
| LNDFH II + LNnDFH II | |  |  |  |  |  |  |  | |
|  | Mean (SD) | 0.07 (0.10) | 0.06 (0.11) |  |  | 0.07 (0.11) | 0.06 (0.11) | 0.162 | |
|  | Median [min, max] | 0.02 [0.01, 0.46] | 0.02 [0.01, 0.73] | 0.14 |  | 0.02 [0.01, 0.58] | 0.02 [0.01, 0.73] |  | |
| Sum of HMOs | |  |  |  |  |  |  |  | |
|  | Mean (SD) | 6.18 (1.41) | 6.27 (1.25) |  |  | 6.16 (1.32) | 6.27 (1.28) | 0.149 | |
|  | Median [min, max] | 6.06 [2.41, 11.6] | 6.21 [2.76, 11.1] | 0.28 |  | 6.04 [2.41, 11.6] | 6.25 [2.76, 11.1] |  | |

P values derived from Wilcoxon sum-rank test comparing HMO concentrations between infants with and without LRTI at 1 year. Bonferroni-adjusted level of statistical significance is α = 0.05/16 = 0.0031. LRTI- Lower respiratory tract infections; HMO, human milk oligosaccharides. 2’-FL, 2’-fucosyllactose; 3-FL, 3-fucosyllactose; 3’-SL, 3’-sialyllactose; 6'-GL, 6’-Galactosyllactose; DFL, 3,2’-difucosyllactose; 6’-SL; 6’-sialyllactose; LNT, lacto-N-tetraose; LNnT, lacto-N-neotetraose; LNFP I, lacto-N-fucopentaose-I; LNFP V, lacto-N-fucopentaose-V; LNFP III, lacto-N-fucopentaose-III; LNFP II, lacto-N-fucopentaose-II; LNDFH I, lacto-N-difucohexaose I; LNDFH II, lacto-N-difucohexaose II; LNnDFH II, lacto-N-neodifucohexaose II

**Table 6.** Absolute human milk oligosaccharide concentrations (g/L) measured at 6 weeks stratified by maternal secretor status and lower respiratory tract infections (LRTI) in infants in the first year of life in the Ulm SPATZ Health Study

|  |  | Secretor milk | |  | Non-secretor milk | |  |
| --- | --- | --- | --- | --- | --- | --- | --- |
|  |  | LRTI Yes (n=111) | LRTI No (n=281) | p | LRTI Yes (n=35) | LRTI No (n=65) | p |
| Lactose | |  |  |  |  |  |  |
|  | Mean (SD) | 66.2 (4.18) | 66.6 (3.60) |  | 67.2 (4.19) | 67.3 (3.41) |  |
|  | Median [min, max] | 67.0 [53.0, 74.0] | 67.0 [52.0, 77.0] | 0.59 | 68.0 [56.0, 74.0] | 68.0 [57.0, 74.0] | 0.77 |
| 2'-FL | |  |  |  |  |  |  |
|  | Mean (SD) | 2.78 (1.04) | 2.83 (0.99) |  | 0.13 (0) | 0.13 (0) |  |
|  | Median [min, max] | 2.60 [0.58, 6.60] | 2.70 [0.13, 6.00] | 0.34 | 0.13 [0.13, 0.13] | 0.13 [0.13, 0.13] |  |
| 3-FL | |  |  |  |  |  |  |
|  | Mean (SD) | 0.46 (0.26) | 0.46 (0.27) |  | 1.41 (0.56) | 1.56 (0.50) |  |
|  | Median [min, max] | 0.41 [0.04, 1.30] | 0.41 [0.03, 1.90] | 0.7 | 1.40 [0.08, 2.60] | 1.60 [0.15, 2.90] | 0.14 |
| 3'-SL | |  |  |  |  |  |  |
|  | Mean (SD) | 0.15 (0.04) | 0.15 (0.04) |  | 0.18 (0.05) | 0.17 (0.06) |  |
|  | Median [min, max] | 0.14 [0.08, 0.27] | 0.14 [0.05, 0.26] | 0.98 | 0.17 [0.08, 0.29] | 0.16 [0.08, 0.32] | 0.34 |
| 6'-GL | |  |  |  |  |  |  |
|  | Mean (SD) | 0.02 (0.01) | 0.02 (0.01) |  | 0.02 (0.01) | 0.02 (0.02) | 0.76 |
|  | Median [min, max] | 0.02 [0.01, 0.05] | 0.02 [0.004, 0.09] | 0.72 | 0.02 [0.01, 0.04] | 0.02 [0.01, 0.15] |  |
| DFL | |  |  |  |  |  |  |
|  | Mean (SD) | 0.24 (0.18) | 0.22 (0.16) |  | 0.01 (0) | 0.01 (0) |  |
|  | Median [min, max] | 0.21 [0.02, 1.40] | 0.20 [0.01, 1.80] | 0.26 | 0.01 [0.01, 0.01] | 0.01 [0.01, 0.01] |  |
| 6'-SL | |  |  |  |  |  |  |
|  | Mean (SD) | 0.26 (0.11) | 0.26 (0.10) |  | 0.26 (0.09) | 0.24 (0.10) |  |
|  | Median [min, max] | 0.24 [0.07, 0.63] | 0.24 [0.06, 0.63] | 0.87 | 0.24 [0.13, 0.55] | 0.23 [0.07, 0.47] | 0.4 |
| LNT | |  |  |  |  |  |  |
|  | Mean (SD) | 0.846 (0.427) | 0.83 (0.40) |  | 1.43 (0.68) | 1.33 (0.56) |  |
|  | Median [min, max] | 0.76 [0.15, 2.60] | 0.77 [0.14, 2.90] | 0.94 | 1.30 [0.36, 3.10] | 1.30 [0.24, 2.40] | 0.66 |
| LNnT | |  |  |  |  |  |  |
|  | Mean (SD) | 0.10 (0.06) | 0.10 (0.06) |  | 0.03 (0.03) | 0.03 (0.02) |  |
|  | Median [min, max] | 0.08 [0.02, 0.30] | 0.09 [0.01, 0.35] | 0.51 | 0.02 [0.01, 0.13] | 0.02 [0.01, 0.10] | 0.81 |
| LNFP I | |  |  |  |  |  |  |
|  | Mean (SD) | 0.61 (0.49) | 0.60 (0.43) |  | 0.04 (0) | 0.04 (0) |  |
|  | Median [min, max] | 0.50 [0.04, 3.00] | 0.51 [0.04, 2.40] | 0.74 | 0.04 [0.04, 0.04] | 0.04 [0.04, 0.04] |  |
| LNFP V | |  |  |  |  |  |  |
|  | Mean (SD) | 0.02 (0.01) | 0.02 (0.01) |  | 0.10 (0.04) | 0.10 (0.05) |  |
|  | Median [min, max] | 0.02 [0.01, 0.08] | 0.02 [0.01, 0.09] | 0.5 | 0.09 [0.01, 0.17] | 0.09 [0.02, 0.24] | 0.75 |
| LNFP III | |  |  |  |  |  |  |
|  | Mean (SD) | 0.18 (0.08) | 0.17 (0.07) |  | 0.21 (0.08) | 0.21 (0.07) |  |
|  | Median [min, max] | 0.17 [0.05, 0.40] | 0.17 [0.04, 0.48] | 0.98 | 0.20 [0.08, 0.43] | 0.20 [0.09, 0.43] | 0.86 |
| LNFP II | |  |  |  |  |  |  |
|  | Mean (SD) | 0.19 (0.15) | 0.19 (0.15) |  | 0.92 (0.39) | 1.00 (0.47) |  |
|  | Median [min, max] | 0.14 [0.04, 0.93] | 0.13 [0.04, 1.20] | 0.65 | 0.93 [0.04, 2.00] | 0.97 [0.04, 2.50] | 0.47 |
| LNDFH I | |  |  |  |  |  |  |
|  | Mean (SD) | 0.69 (0.32) | 0.66 (0.32) |  | 0.02 (0) | 0.02 (0) |  |
|  | Median [min, max] | 0.68 [0.02, 1.70] | 0.63 [0.02, 1.90] | 0.18 | 0.02 [0.02, 0.02] | 0.02 [0.02, 0.02] |  |
| LNDFH II + LNnDFH II | |  |  |  |  |  |  |
|  | Mean (SD) | 0.02 (0.02) | 0.02 (0.02) |  | 0.21 (0.12) | 0.25 (0.15) |  |
|  | Median [min, max] | 0.01 [0.01, 0.11] | 0.01 [0.01, 0.18] | 0.31 | 0.18 [0.010, 0.46] | 0.23 [0.01, 0.73] | 0.2 |
| Sum of HMOs | |  |  |  |  |  |  |
|  | Mean (SD) | 6.56 (1.30) | 6.53 (1.12) |  | 4.96 (0.98) | 5.12 (1.17) |  |
|  | Median [min, max] | 6.49 [3.78, 11.6] | 6.41 [3.58, 11.1] | 0.91 | 4.92 [2.41, 7.20] | 4.99 [2.76, 8.40] | 0.65 |

P values derived from Wilcoxon sum-rank test comparing HMO concentrations between infants with and without LRTI at 1 year within the secretor and non-secretor milk groups. Bonferroni-adjusted level of statistical significance is α = 0.05/16 = 0.0031. LRTI- Lower respiratory tract infections; HMO, human milk oligosaccharides. 2’-FL, 2’-fucosyllactose; 3-FL, 3-fucosyllactose; 3’-SL, 3’-sialyllactose; 6'-GL, 6’-Galactosyllactose; DFL, 3,2’-difucosyllactose; 6’-SL; 6’-sialyllactose; LNT, lacto-N-tetraose; LNnT, lacto-N-neotetraose; LNFP I, lacto-N-fucopentaose-I; LNFP V, lacto-N-fucopentaose-V; LNFP III, lacto-N-fucopentaose-III; LNFP II, lacto-N-fucopentaose-II; LNDFH I, lacto-N-difucohexaose I; LNDFH II, lacto-N-difucohexaose II; LNnDFH II, lacto-N-neodifucohexaose II

**Table 7.** Absolute human milk oligosaccharide concentrations (g/L) measured at 6 weeks stratified by maternal secretor status and lower respiratory tract infections (LRTI) in infants in the second year of life in the Ulm SPATZ Health Study

|  |  | Secretor milk | |  | Non-secretor milk | |  |
| --- | --- | --- | --- | --- | --- | --- | --- |
|  |  | LRTI Yes (n=187) | LRTI No (n=245) | p | LRTI Yes (n=52) | LRTI No (n=52) | p |
| Lactose | |  |  |  |  |  |  |
|  | Mean (SD) | 66.4 (3.85) | 66.6 (3.64) |  | 67.0 (3.94) | 67.5 (3.33) |  |
|  | Median [min, max] | 67.0 [53.0, 74.0] | 67.0 [52.0, 77.0] | 0.65 | 67.5 [56.0, 74.0] | 68.0 [57.0, 74.0] | 0.784 |
| 2'-FL | |  |  |  |  |  |  |
|  | Mean (SD) | 2.76 (1.03) | 2.85 (0.98) |  | 0.13 (0) | 0.13 (0) |  |
|  | Median [min, max] | 2.60 [0.13, 6.60] | 2.80 [0.31, 5.90] | 0.18 | 0.13 [0.13, 0.13] | 0.13 [0.13, 0.13] |  |
| 3-FL | |  |  |  |  |  |  |
|  | Mean (SD) | 0.46 (0.28) | 0.45 (0.25) |  | 1.52 (0.56) | 1.47 (0.51) |  |
|  | Median [min, max] | 0.40 [0.03, 1.90] | 0.41 [0.03, 1.50] | 0.99 | 1.50 [0.08, 2.90] | 1.60 [0.15, 2.40] | 0.822 |
| 3'-SL | |  |  |  |  |  |  |
|  | Mean (SD) | 0.14 (0.04) | 0.15 (0.04) |  | 0.18 (0.05) | 0.17 (0.05) |  |
|  | Median [min, max] | 0.14 [0.05, 0.27] | 0.14 [0.07, 0.26] | 0.45 | 0.18 [0.08, 0.30] | 0.15 [0.07, 0.32] | 0.015 |
| 6'-GL | |  |  |  |  |  |  |
|  | Mean (SD) | 0.02 (0.01) | 0.02 (0.01) |  | 0.02 (0.01) | 0.02 (0.02) |  |
|  | Median [min, max] | 0.02 [0.004, 0.05] | 0.02 [0.01, 0.09] | 0.64 | 0.02 [0.01, 0.04] | 0.02 [0.01, 0.15] | 0.274 |
| DFL | |  |  |  |  |  |  |
|  | Mean (SD) | 0.22 (0.15) | 0.23 (0.17) |  | 0.01 (0) | 0.01 (0) |  |
|  | Median [min, max] | 0.20 [0.01, 1.40] | 0.20 [0.02, 1.80] | 0.96 | 0.01 [0.01, 0.01] | 0.01 [0.01, 0.01] |  |
| 6'-SL | |  |  |  |  |  |  |
|  | Mean (SD) | 0.26 (0.11) | 0.26 (0.10) |  | 0.26 (0.10) | 0.24 (0.10) |  |
|  | Median [min, max] | 0.23 [0.07, 0.73] | 0.25 [0.05, 0.63] | 0.34 | 0.25 [0.07, 0.55] | 0.22 [0.08, 0.47] | 0.079 |
| LNT | |  |  |  |  |  |  |
|  | Mean (SD) | 0.85 (0.40) | 0.83 (0.40) |  | 1.35 (0.66) | 1.36 (0.54) |  |
|  | Median [min, max] | 0.79 [0.15, 2.60] | 0.76 [0.14, 2.90] | 0.69 | 1.20 [0.24, 3.10] | 1.30 [0.51, 2.30] | 0.73 |
| LNnT | |  |  |  |  |  |  |
|  | Mean (SD) | 0.10 (0.06) | 0.10 (0.06) |  | 0.03 (0.03) | 0.03 (0.02) |  |
|  | Median [min, max] | 0.08 [0.02, 0.35] | 0.09 [0.01, 0.330] | 0.46 | 0.02 [0.01, 0.13] | 0.02 [0.01, 0.09] | 0.837 |
| LNFP I | |  |  |  |  |  |  |
|  | Mean (SD) | 0.60 (0.45) | 0.61 (0.45) |  | 0.04 (0) | 0.04 (0) |  |
|  | Median [min, max] | 0.50 [0.04, 3.00] | 0.51 [0.04, 2.70] | 0.72 | 0.04 [0.04, 0.04] | 0.04 [0.04, 0.04] |  |
| LNFP V | |  |  |  |  |  |  |
|  | Mean (SD) | 0.02 (0.01) | 0.02 (0.01) |  | 0.10 (0.04) | 0.10 (0.05) |  |
|  | Median [min, max] | 0.02 [0.01, 0.08] | 0.02 [0.01, 0.09] | 0.58 | 0.09 [0.01, 0.17] | 0.09 [0.02, 0.24] | 0.958 |
| LNFP III | |  |  |  |  |  |  |
|  | Mean (SD) | 0.17 (0.07) | 0.18 (0.07) |  | 0.20 (0.07) | 0.22 (0.07) |  |
|  | Median [min, max] | 0.16 [0.05, 0.40] | 0.17 [0.04, 0.48] | 0.27 | 0.19 [0.08, 0.43] | 0.21 [0.09, 0.43] | 0.319 |
| LNFP II | |  |  |  |  |  |  |
|  | Mean (SD) | 0.19 (0.15) | 0.19 (0.15) |  | 0.96 (0.40) | 0.10 (0.49) |  |
|  | Median [min, max] | 0.15 [0.04, 0.93] | 0.13 [0.04, 1.20] | 0.65 | 0.94 [0.04, 2.00] | 0.92 [0.04, 2.50] | 0.987 |
| LNDFH I | |  |  |  |  |  |  |
|  | Mean (SD) | 0.67 (0.32) | 0.66 (0.32) |  | 0.02 (0) | 0.02 (0) |  |
|  | Median [min, max] | 0.65 [0.02, 1.70] | 0.63 [0.02, 1.90] | 0.34 | 0.02 [0.02, 0.02] | 0.02 [0.02, 0.02] |  |
| LNDFH II + LNnDFH II | |  |  |  |  |  |  |
|  | Mean (SD) | 0.02 (0.02) | 0.02 (0.02) |  | 0.23 (0.13) | 0.24 (0.15) |  |
|  | Median [min, max] | 0.01 [0.01, 0.18] | 0.01 [0.01, 0.15] | 0.63 | 0.20 [0.01, 0.58] | 0.23 [0.01, 0.73] | 0.943 |
| Sum of HMOs | |  |  |  |  |  |  |
|  | Mean (SD) | 6.47 (1.22) | 6.54 (1.12) |  | 5.07 (1.04) | 4.99 (1.19) |  |
|  | Median [min, max] | 6.28 [3.78, 11.6] | 6.46 [3.58, 11.1] | 0.27 | 5.03 [2.41, 7.58] | 4.79 [2.76, 8.40] | 0.603 |

P values derived from Wilcoxon sum-rank test comparing HMO concentrations between infants with and without LRTI at 1 year within the secretor and non-secretor milk groups. Bonferroni-adjusted level of statistical significance is α = 0.05/16 = 0.0031. LRTI- Lower respiratory tract infections; HMO, human milk oligosaccharides. 2’-FL, 2’-fucosyllactose; 3-FL, 3-fucosyllactose; 3’-SL, 3’-sialyllactose; 6'-GL, 6’-Galactosyllactose; DFL, 3,2’-difucosyllactose; 6’-SL; 6’-sialyllactose; LNT, lacto-N-tetraose; LNnT, lacto-N-neotetraose; LNFP I, lacto-N-fucopentaose-I; LNFP V, lacto-N-fucopentaose-V; LNFP III, lacto-N-fucopentaose-III; LNFP II, lacto-N-fucopentaose-II; LNDFH I, lacto-N-difucohexaose I; LNDFH II, lacto-N-difucohexaose II; LNnDFH II, lacto-N-neodifucohexaose II

**Table 8**: Absolute human milk oligosaccharide concentrations (g/L) measured at 6 weeks stratified by maternal milk group and lower respiratory tract infections (LRTI) in infants in the first year of life in the Ulm SPATZ Health Study

|  |  | Group I milk | |  | Group II milk | |  |
| --- | --- | --- | --- | --- | --- | --- | --- |
|  |  | LRTI Yes (n=102) | LRTI No (n=260) | p | LRTI Yes (n=34) | LRTI No (n=62) | p |
| Lactose | |  |  |  |  |  |  |
|  | Mean (SD) | 66.2 (4.14) | 66.5 (3.64) |  | 67.1 (4.22) | 67.3 (3.48) |  |
|  | Median [min, max] | 66.5 [53.0, 74.0] | 67.0 [52.0, 77.0] | 0.68 | 68.0 [56.0, 74.0] | 68.0 [57.0, 74.0] | 0.899 |
| 2'-FL | |  |  |  |  |  |  |
|  | Mean (SD) | 2.64 (0.90) | 2.71 (0.90) |  | 0.13 (0) | 0.13 (0) |  |
|  | Median [min, max] | 2.50 [0.58, 6.30] | 2.70 [0.13, 5.60] | 0.29 | 0.13 [0.13, 0.13] | 0.13 [0.13, 0.13] |  |
| 3-FL | |  |  |  |  |  |  |
|  | Mean (SD) | 0.50 (0.24) | 0.49 (0.26) |  | 1.45 (0.52) | 1.62 (0.42) |  |
|  | Median [min, max] | 0.42 [0.14, 1.30] | 0.43 [0.09, 1.90] | 0.6 | 1.40 [0.63, 2.60] | 1.60 [0.84, 2.90] | 0.088 |
| 3'-SL | |  |  |  |  |  |  |
|  | Mean (SD) | 0.15 (0.04) | 0.15 (0.04) |  | 0.18 (0.05) | 0.17 (0.06) |  |
|  | Median [min, max] | 0.14 [0.08, 0.27] | 0.14 [0.05, 0.26] | 0.79 | 0.18 [0.11, 0.29] | 0.16 [0.07, 0.32] | 0.229 |
| 6'-GL | |  |  |  |  |  |  |
|  | Mean (SD) | 0.02 (0.01) | 0.02 (0.01) |  | 0.02 (0.01) | 0.02 (0.02) |  |
|  | Median [min, max] | 0.02 [0.01, 0.05] | 0.02 [0.004, 0.09] | 0.61 | 0.02 [0.01, 0.04] | 0.02 [0.01, 0.15] | 0.824 |
| DFL | |  |  |  |  |  |  |
|  | Mean (SD) | 0.25 (0.17) | 0.24 (0.16) |  | 0.01 (0) | 0.01 (0) |  |
|  | Median [min, max] | 0.22 [0.07, 1.40] | 0.20 [0.05, 1.80] | 0.25 | 0.01 [0.01, 0.01] | 0.01 [0.01, 0.01] |  |
| 6'-SL | |  |  |  |  |  |  |
|  | Mean (SD) | 0.26 (0.11) | 0.26 (0.10) |  | 0.26 (0.10) | 0.24 (0.10) |  |
|  | Median [min, max] | 0.24 [0.07, 0.63] | 0.24 [0.06, 0.61] | 0.97 | 0.24 [0.13, 0.55] | 0.23 [0.07, 0.47] | 0.388 |
| LNT | |  |  |  |  |  |  |
|  | Mean (SD) | 0.86 (0.43) | 0.85 (0.39) |  | 1.42 (0.69) | 1.29 (0.55) |  |
|  | Median [min, max] | 0.84 [0.15, 2.60] | 0.80 [0.14, 2.90] | 0.99 | 1.30 [0.36, 3.10] | 1.20 [0.24, 2.40] | 0.481 |
| LNnT | |  |  |  |  |  |  |
|  | Mean (SD) | 0.10 (0.06) | 0.10 (0.06) |  | 0.03 (0.03) | 0.03 (0.02) |  |
|  | Median [min, max] | 0.09 [0.02, 0.30] | 0.09 [0.01, 0.35] | 0.61 | 0.02 [0.01, 0.13] | 0.02 [0.01, 0.10] | 0.89 |
| LNFP I | |  |  |  |  |  |  |
|  | Mean (SD) | 0.54 (0.40) | 0.55 (0.37) |  | 0.04 (0) | 0.04 (0) |  |
|  | Median [min, max] | 0.42 [0.04, 1.80] | 0.47 [0.04, 1.90] | 0.57 | 0.04 [0.04, 0.04] | 0.04 [0.04, 0.04] |  |
| LNFP V | |  |  |  |  |  |  |
|  | Mean (SD) | 0.02 (0.01) | 0.02 (0.01) |  | 0.10 (0.04) | 0.10 (0.04) |  |
|  | Median [min, max] | 0.02 [0.01, 0.08] | 0.02 [0.01, 0.09] | 0.46 | 0.09 [0.03, 0.17] | 0.09 [0.03, 0.24] | 0.581 |
| LNFP III | |  |  |  |  |  |  |
|  | Mean (SD) | 0.18 (0.08) | 0.18 (0.07) |  | 0.21 (0.08) | 0.21 (0.07) |  |
|  | Median [min, max] | 0.17 [0.05, 0.40] | 0.18 [0.04, 0.48] | 0.83 | 0.20 [0.08, 0.43] | 0.20 [0.09, 0.43] | 0.768 |
| LNFP II | |  |  |  |  |  |  |
|  | Mean (SD) | 0.21 (0.15) | 0.20 (0.15) |  | 0.95 (0.37) | 1.05 (0.43) |  |
|  | Median [min, max] | 0.16 [0.04, 0.93] | 0.15 [0.04, 1.20] | 0.55 | 0.94 [0.24, 2.00] | 0.98 [0.34, 2.50] | 0.355 |
| LNDFH I | |  |  |  |  |  |  |
|  | Mean (SD) | 0.75 (0.27) | 0.71 (0.28) |  | 0.02 (0) | 0.02 (0) |  |
|  | Median [min, max] | 0.70 [0.22, 1.70] | 0.65 [0.07, 1.90] | 0.1 | 0.02 [0.02, 0.02] | 0.02 [0.02, 0.02] |  |
| LNDFH II + LNnDFH II | |  |  |  |  |  |  |
|  | Mean (SD) | 0.02 (0.02) | 0.02 (0.02) |  | 0.22 (0.12) | 0.26 (0.14) |  |
|  | Median [min, max] | 0.01 [0.01, 0.11] | 0.01 [0.01, 0.18] | 0.27 | 0.19 [0.07, 0.46] | 0.23 [0.04, 0.73] | 0.132 |
| Sum of HMOs | |  |  |  |  |  |  |
|  | Mean (SD) | 6.50 (1.23) | 6.49 (1.10) |  | 5.04 (0.89) | 5.19 (1.14) |  |
|  | Median [min, max] | 6.46 [3.78, 10.7] | 6.36 [3.58, 11.1] | 0.98 | 4.95 [3.39, 7.20] | 5.07 [2.76, 8.40] | 0.563 |

P values derived from Wilcoxon sum-rank test comparing HMO concentrations between infants with and without LRTI at 1 year within group I and group II milk. Bonferroni-adjusted level of statistical significance is α = 0.05/16 = 0.0031. LRTI- Lower respiratory tract infections; HMO, human milk oligosaccharides. 2’-FL, 2’-fucosyllactose; 3-FL, 3-fucosyllactose; 3’-SL, 3’-sialyllactose; 6'-GL, 6’-Galactosyllactose; DFL, 3,2’-difucosyllactose; 6’-SL; 6’-sialyllactose; LNT, lacto-N-tetraose; LNnT, lacto-N-neotetraose; LNFP I, lacto-N-fucopentaose-I; LNFP V, lacto-N-fucopentaose-V; LNFP III, lacto-N-fucopentaose-III; LNFP II, lacto-N-fucopentaose-II; LNDFH I, lacto-N-difucohexaose I; LNDFH II, lacto-N-difucohexaose II; LNnDFH II, lacto-N-neodifucohexaose II

**Table 9**: Absolute human milk oligosaccharide concentrations (g/L) measured at 6 weeks stratified by maternal milk group and lower respiratory tract infections (LRTI) in infants in the second year of life in the Ulm SPATZ Health Study

|  |  | Group I milk | |  | Group II milk | |  |
| --- | --- | --- | --- | --- | --- | --- | --- |
|  |  | LRTI Yes (n=171) | LRTI No (n=228) | p | LRTI Yes (n=51) | LRTI No (n=48) | p |
| Lactose | |  |  |  |  |  |  |
|  | Mean (SD) | 66.4 (3.79) | 66.6 (3.63) |  | 67.0 (3.96) | 67.4 (3.45) |  |
|  | Median [min, max] | 67.0 [53.0, 74.0] | 67.0 [52.0, 77.0] | 0.62 | 67.0 [56.0, 74.0] | 68.0 [57.0, 74.0] | 0.714 |
| 2'-FL | |  |  |  |  |  |  |
|  | Mean (SD) | 2.64 (0.90) | 2.74 (0.89) |  | 0.13 (0) | 0.13 (0) |  |
|  | Median [min, max] | 2.50 [0.13, 6.30] | 2.70 [0.31, 5.60] | 0.15 | 0.13 [0.13, 0.13] | 0.13 [0.13, 0.13] |  |
| 3-FL | |  |  |  |  |  |  |
|  | Mean (SD) | 0.49 (0.26) | 0.48 (0.24) |  | 1.55 (0.53) | 1.57 (0.39) |  |
|  | Median [min, max] | 0.42 [0.09, 1.90] | 0.43 [0.10, 1.50] | 0.74 | 1.50 [0.63, 2.90] | 1.60 [0.84, 2.40] | 0.776 |
| 3'-SL | |  |  |  |  |  |  |
|  | Mean (SD) | 0.14 (0.04) | 0.15 (0.04) |  | 0.19 (0.05) | 0.17 (0.05) |  |
|  | Median [min, max] | 0.14 [0.05, 0.27] | 0.14 [0.07, 0.26] | 0.67 | 0.18 [0.11, 0.30] | 0.15 [0.07, 0.32] | 0.011 |
| 6'-GL | |  |  |  |  |  |  |
|  | Mean (SD) | 0.02 (0.01) | 0.02 (0.01) |  | 0.02 (0.01) | 0.02 (0.02) |  |
|  | Median [min, max] | 0.02 [0.004, 0.05] | 0.02 [0.01, 0.09] | 0.71 | 0.02 [0.01, 0.04] | 0.02 [0.01, 0.15] | 0.192 |
| DFL | |  |  |  |  |  |  |
|  | Mean (SD) | 0.23 (0.15) | 0.24 (0.17) |  | 0.01 (0) | 0.01 (0) |  |
|  | Median [min, max] | 0.21 [0.07, 1.40] | 0.20 [0.05, 1.80] | 0.85 | 0.01 [0.01, 0.01] | 0.01 [0.01, 0.01] |  |
| 6'-SL | |  |  |  |  |  |  |
|  | Mean (SD) | 0.25 (0.10) | 0.26 (0.10) |  | 0.26 (0.10) | 0.23 (0.11) |  |
|  | Median [min, max] | 0.23 [0.07, 0.63] | 0.24 [0.05, 0.61] | 0.34 | 0.25 [0.07, 0.56] | 0.21 [0.08, 0.47] | 0.059 |
| LNT | |  |  |  |  |  |  |
|  | Mean (SD) | 0.86 (0.40) | 0.83 (0.40) |  | 1.35 (0.66) | 1.31 (0.53) |  |
|  | Median [min, max] | 0.85 [0.15, 2.60] | 0.77 [0.14, 2.90] | 0.5 | 1.20 [0.24, 3.10] | 1.30 [0.51, 2.30] | 0.947 |
| LNnT | |  |  |  |  |  |  |
|  | Mean (SD) | 0.10 (0.07) | 0.10 (0.06) |  | 0.03 (0.03) | 0.02 (0.02) |  |
|  | Median [min, max] | 0.09 [0.02, 0.35] | 0.09 [0.01, 0.33] | 0.66 | 0.02 [0.01, 0.13] | 0.02 [0.01, 0.09] | 0.877 |
| LNFP I | |  |  |  |  |  |  |
|  | Mean (SD) | 0.55 (0.40) | 0.55 (0.36) |  | 0.04 (0) | 0.040 (0) |  |
|  | Median [min, max] | 0.44 [0.04, 1.80] | 0.47 [0.04, 1.90] | 0.68 | 0.04 [0.04, 0.04] | 0.04 [0.04, 0.04] |  |
| LNFP V | |  |  |  |  |  |  |
|  | Mean (SD) | 0.02 (0.01) | 0.02 (0.01) |  | 0.10 (0.04) | 0.10 (0.04) |  |
|  | Median [min, max] | 0.02 [0.01, 0.08] | 0.02 [0.01, 0.09] | 0.38 | 0.09 [0.03, 0.17] | 0.09 [0.03, 0.24] | 0.936 |
| LNFP III | |  |  |  |  |  |  |
|  | Mean (SD) | 0.17 (0.07) | 0.18 (0.07) |  | 0.21 (0.07) | 0.21 (0.07) |  |
|  | Median [min, max] | 0.17 [0.05, 0.40] | 0.18 [0.04, 0.48] | 0.34 | 0.19 [0.08, 0.43] | 0.20 [0.09, 0.43] | 0.614 |
| LNFP II | |  |  |  |  |  |  |
|  | Mean (SD) | 0.21 (0.15) | 0.20 (0.15) |  | 0.98 (0.38) | 1.04 (0.43) |  |
|  | Median [min, max] | 0.16 [0.04, 0.93] | 0.14 [0.04, 1.20] | 0.41 | 0.95 [0.24, 2.00] | 0.97 [0.34, 2.50] | 0.602 |
| LNDFH I | |  |  |  |  |  |  |
|  | Mean (SD) | 0.73 (0.27) | 0.70 (0.27) |  | 0.02 (0) | 0.02 (0) |  |
|  | Median [min, max] | 0.67 [0.07, 1.70] | 0.65 [0.14, 1.90] | 0.16 | 0.02 [0.02, 0.02] | 0.02 [0.02, 0.02] |  |
| LNDFH II + LNnDFH II | |  |  |  |  |  |  |
|  | Mean (SD) | 0.02 (0.02) | 0.02 (0.02) |  | 0.24 (0.13) | 0.26 (0.14) |  |
|  | Median [min, max] | 0.01 [0.01, 0.18] | 0.01 [0.01, 0.15] | 0.49 | 0.20 [0.07, 0.58] | 0.230 [0.04, 0.73] | 0.535 |
| Sum of HMOs | |  |  |  |  |  |  |
|  | Mean (SD) | 6.44 (1.17) | 6.47 (1.08) |  | 5.12 (0.98) | 5.12 (1.13) |  |
|  | Median [min, max] | 6.26 [3.78, 10.7] | 6.41 [3.58, 11.1] | 0.49 | 5.03 [3.39, 7.58] | 4.87 [2.76, 8.40] | 0.927 |

P values derived from Wilcoxon sum-rank test comparing HMO concentrations between infants with and without LRTI at 1 year within milk groups I and II. Bonferroni-adjusted level of statistical significance is α = 0.05/16 = 0.0031. LRTI- Lower respiratory tract infections; HMO, human milk oligosaccharides. 2’-FL, 2’-fucosyllactose; 3-FL, 3-fucosyllactose; 3’-SL, 3’-sialyllactose; 6'-GL, 6’-Galactosyllactose; DFL, 3,2’-difucosyllactose; 6’-SL; 6’-sialyllactose; LNT, lacto-N-tetraose; LNnT, lacto-N-neotetraose; LNFP I, lacto-N-fucopentaose-I; LNFP V, lacto-N-fucopentaose-V; LNFP III, lacto-N-fucopentaose-III; LNFP II, lacto-N-fucopentaose-II; LNDFH I, lacto-N-difucohexaose I; LNDFH II, lacto-N-difucohexaose II; LNnDFH II, lacto-N-neodifucohexaose II

**Table 10**. Absolute human milk oligosaccharide concentrations (g/L) measured at 6 weeks and upper respiratory tract infections (URTI) in infants in the first or second year of life in the Ulm SPATZ Health Study

|  |  | URTI in the first year of life | |  | URTI in the second year of life | |  |
| --- | --- | --- | --- | --- | --- | --- | --- |
|  |  | URTI Yes (n=326) | URTI No (n=166) | p | URTI Yes (n=433) | URTI No (n=102) | p |
| Lactose | |  |  |  |  |  |  |
|  | Mean (SD) | 66.8 (3.76) | 66.3 (3.78) |  | 66.7 (3.76) | 66.5 (3.63) |  |
|  | Median [min, max] | 67.0 [53.0, 74.0] | 67.0 [52.0, 77.0] | 0.086 | 67.0 [52.0, 77.0] | 67.0 [53.0, 74.0] | 0.718 |
| 2'-FL | |  |  |  |  |  |  |
|  | Mean (SD) | 2.27 (1.39) | 2.28 (1.45) |  | 2.25 (1.39) | 2.51 (1.38) |  |
|  | Median [min, max] | 2.40 [0.13, 6.60] | 2.50 [0.13, 5.60] | 0.743 | 2.40 [0.13, 6.60] | 2.60 [0.13, 5.60] | 0.105 |
| 3-FL | |  |  |  |  |  |  |
|  | Mean (SD) | 0.66 (0.54) | 0.68 (0.56) |  | 0.67 (0.54) | 0.59 (0.48) |  |
|  | Median [min, max] | 0.49 [0.039, 2.60] | 0.49 [0.03, 2.90] | 0.936 | 0.49 [0.03, 2.90] | 0.44 [0.03, 2.40] | 0.175 |
| 3'-SL | |  |  |  |  |  |  |
|  | Mean (SD) | 0.15 (0.04) | 0.15 (0.04) |  | 0.15 (0.04) | 0.15 (0.04) |  |
|  | Median [min, max] | 0.15 [0.07, 0.30] | 0.15 [0.07, 0.32] | 0.604 | 0.15 [0.05, 0.32] | 0.15 [0.08, 0.27] | 0.765 |
| 6'-GL | |  |  |  |  |  |  |
|  | Mean (SD) | 0.02 (0.01) | 0.02 (0.01) |  | 0.02 (0.01) | 0.02 (0.01) |  |
|  | Median [min, max] | 0.02 [0.004, 0.15] | 0.02 [0.01, 0.09] | 0.947 | 0.02 [0.004, 0.15] | 0.02 [0.001, 0.04] | 0.737 |
| DFL | |  |  |  |  |  |  |
|  | Mean (SD) | 0.18 (0.15) | 0.19 (0.21) |  | 0.18 (0.17) | 0.19 (0.15) |  |
|  | Median [min, max] | 0.17 [0.01, 1.40] | 0.17 [0.01, 1.80] | 0.8 | 0.17 [0.01, 1.80] | 0.20 [0.01, 0.88] | 0.187 |
| 6'-SL | |  |  |  |  |  |  |
|  | Mean (SD) | 0.26 (0.10) | 0.26 (0.11) |  | 0.26 (0.11) | 0.26 (0.10) |  |
|  | Median [min, max] | 0.24 [0.08, 0.73] | 0.25 [0.06, 0.6] | 0.831 | 0.24 [0.05, 0.73] | 0.24 [0.07, 0.54] | 0.693 |
| LNT | |  |  |  |  |  |  |
|  | Mean (SD) | 0.94 (0.50) | 0.95 (0.50) |  | 0.94 (0.51) | 0.92 (0.42) |  |
|  | Median [min, max] | 0.85 [0.14, 3.10] | 0.85 [0.14, 2.90] | 0.874 | 0.84 [0.14, 3.10] | 0.85 [0.19, 2.20] | 0.846 |
| LNnT | |  |  |  |  |  |  |
|  | Mean (SD) | 0.09 (0.06) | 0.08 (0.07) |  | 0.08 (0.06) | 0.09 (0.06) |  |
|  | Median [min, max] | 0.07 [0.01, 0.33] | 0.08 [0.01, 0.35] | 0.851 | 0.07 [0.01, 0.35] | 0.08 [0.01, 0.24] | 0.375 |
| LNFP I | |  |  |  |  |  |  |
|  | Mean (SD) | 0.48 (0.45) | 0.52 (0.47) |  | 0.47 (0.44) | 0.60 (0.52) |  |
|  | Median [min, max] | 0.38 [0.04, 3.00] | 0.40 [0.04, 1.90] | 0.618 | 0.36 [0.04, 3.00] | 0.48 [0.04, 2.70] | 0.025 |
| LNFP V | |  |  |  |  |  |  |
|  | Mean (SD) | 0.04 (0.04) | 0.04 (0.04) |  | 0.04 (0.04) | 0.03 (0.04) |  |
|  | Median [min, max] | 0.02 [0.01, 0.21] | 0.02 [0.01, 0.24] | 0.694 | 0.02 [0.01, 0.21] | 0.02 [0.01, 0.24] | 0.348 |
| LNFP III | |  |  |  |  |  |  |
|  | Mean (SD) | 0.18 (0.07) | 0.18 (0.08) |  | 0.18 (0.07) | 0.18 (0.08) |  |
|  | Median [min, max] | 0.18 [0.05, 0.48] | 0.18 [0.04, 0.43] | 0.803 | 0.18 [0.04, 0.48] | 0.17 [0.05, 0.43] | 0.525 |
| LNFP II | |  |  |  |  |  |  |
|  | Mean (SD) | 0.35 (0.40) | 0.35 (0.40) |  | 0.35 (0.40) | 0.29 (0.33) |  |
|  | Median [min, max] | 0.17 [0.04, 2.00] | 0.21 [0.04, 2.50] | 0.648 | 0.17 [0.04, 2.50] | 0.20 [0.04, 1.70] | 0.514 |
| LNDFH I | |  |  |  |  |  |  |
|  | Mean (SD) | 0.53 (0.38) | 0.54 (0.40) |  | 0.52 (0.38) | 0.58 (0.38) |  |
|  | Median [min, max] | 0.58 [0.02, 1.70] | 0.58 [0.02, 1.90] | 0.655 | 0.57 [0.02, 1.90] | 0.61 [0.02, 1.70] | 0.152 |
| LNDFH II + LNnDFH II | |  |  |  |  |  |  |
|  | Mean (SD) | 0.06 (0.10) | 0.07 (0.12) |  | 0.07 (0.11) | 0.05 (0.10) |  |
|  | Median [min, max] | 0.02 [0.01, 0.49] | 0.02 [0.01, 0.73] | 0.726 | 0.02 [0.02, 0.73] | 0.02 [0.01, 0.70] | 0.873 |
| Sum of HMOs | |  |  |  |  |  |  |
|  | Mean (SD) | 6.20 (1.23) | 6.30 (1.43) |  | 6.17 (1.28) | 6.45 (1.34) |  |
|  | Median [min, max] | 6.11 [2.41, 11.6] | 6.36 [2.76, 11.1] | 0.32 | 6.10 [2.41, 11.6] | 6.60 [3.39, 9.80] | 0.021 |

P values derived from Wilcoxon sum-rank test comparing HMO concentrations between infants with and without URTI at 1 year. Bonferroni-adjusted level of statistical significance is α = 0.05/16 = 0.0031. URTI- Upper respiratory tract infections; HMO, human milk oligosaccharides. 2’-FL, 2’-fucosyllactose; 3-FL, 3-fucosyllactose; 3’-SL, 3’-sialyllactose; 6'-GL, 6’-Galactosyllactose; DFL, 3,2’-difucosyllactose; 6’-SL; 6’-sialyllactose; LNT, lacto-N-tetraose; LNnT, lacto-N-neotetraose; LNFP I, lacto-N-fucopentaose-I; LNFP V, lacto-N-fucopentaose-V; LNFP III, lacto-N-fucopentaose-III; LNFP II, lacto-N-fucopentaose-II; LNDFH I, lacto-N-difucohexaose I; LNDFH II, lacto-N-difucohexaose II; LNnDFH II, Lacto-N-neodifucohexaose II.

**Table 11**. Absolute human milk oligosaccharide concentrations (g/L) measured at 6 weeks stratified by maternal secretor status and upper respiratory tract infections (URTI) in infants in the first year of life in the Ulm SPATZ Health Study

|  |  | Secretor milk | |  | Non-secretor milk | |  |
| --- | --- | --- | --- | --- | --- | --- | --- |
|  |  | URTI Yes (n=263) | URTI No (n=129) | p | URTI Yes (n=63) | URTI No (n=37) | p |
| Lactose | |  |  |  |  |  |  |
|  | Mean (SD) | 66.6 (3.74) | 66.1 (3.86) |  | 67.6 (3.78) | 66.8 (3.52) |  |
|  | Median [min, max] | 67.0 [53.0, 74.0] | 67.0 [52.0, 77.0] | 0.157 | 69.0 [58.0, 74.0] | 67.0 [56.0, 72.0] | 0.168 |
| 2'-FL | |  |  |  |  |  |  |
|  | Mean (SD) | 2.78 (1.01) | 2.90 (1.00) |  | 0.13 (0) | 0.13 (0) |  |
|  | Median [min, max] | 2.70 [0.31, 6.60] | 2.80 [0.13, 5.60] | 0.21 | 0.13 [0.13, 0.13] | 0.13 [0.13, 0.13] |  |
| 3-FL | |  |  |  |  |  |  |
|  | Mean (SD) | 0.46 (0.27) | 0.44 (0.27) |  | 1.51 (0.54) | 1.51 (0.50) |  |
|  | Median [min, max] | 0.41 [0.03, 1.50] | 0.39 [0.03, 1.90] | 0.406 | 1.60 [0.08, 2.60] | 1.50 [0.63, 2.90] | 0.601 |
| 3'-SL | |  |  |  |  |  |  |
|  | Mean (SD) | 0.15 (0.04) | 0.15 (0.03) |  | 0.18 (0.05) | 0.18 (0.06) |  |
|  | Median [min, max] | 0.14 [0.07, 0.26] | 0.15 [0.08, 0.27] | 0.566 | 0.17 [0.08, 0.30] | 0.17 [0.07, 0.32] | 0.897 |
| 6'-GL | |  |  |  |  |  |  |
|  | Mean (SD) | 0.02 (0.01) | 0.02 (0.01) |  | 0.02 (0.02) | 0.02 (0.01) |  |
|  | Median [min, max] | 0.02 [0.004, 0.05] | 0.02 [0.01, 0.09] | 0.393 | 0.02 [0.01, 0.15] | 0.02 [0.01, 0.03] | 0.091 |
| DFL | |  |  |  |  |  |  |
|  | Mean (SD) | 0.22 (0.14) | 0.24 (0.21) |  | 0.01 (0) | 0.01 (0) |  |
|  | Median [min, max] | 0.20 [0.02, 1.40] | 0.21 [0.01, 1.80] | 0.664 | 0.01 [0.01, 0.01] | 0.01 [0.01, 0.01] |  |
| 6'-SL | |  |  |  |  |  |  |
|  | Mean (SD) | 0.26 (0.11) | 0.26 (0.10) |  | 0.25 (0.09) | 0.25 (0.11) |  |
|  | Median [min, max] | 0.24 [0.07, 0.73] | 0.25 [0.06, 0.61] | 0.934 | 0.24 [0.08, 0.47] | 0.23 [0.07, 0.55] | 0.783 |
| LNT | |  |  |  |  |  |  |
|  | Mean (SD) | 0.84 (0.42) | 0.84 (0.40) |  | 1.38 (0.61) | 1.35 (0.61) |  |
|  | Median [min, max] | 0.79 [0.14, 2.60] | 0.76 [0.14, 2.90] | 0.915 | 1.20 [0.36, 3.10] | 1.30 [0.24, 2.60] | 0.92 |
| LNnT | |  |  |  |  |  |  |
|  | Mean (SD) | 0.10 (0.06) | 0.10 (0.06) |  | 0.03 (0.02) | 0.02 (0.02) |  |
|  | Median [min, max] | 0.09 [0.01, 0.33] | 0.09 [0.02, 0.35] | 0.366 | 0.02 [0.01, 0.10] | 0.02 [0.01, 0.13] | 0.027 |
| LNFP I | |  |  |  |  |  |  |
|  | Mean (SD) | 0.59 (0.44) | 0.65 (0.44) |  | 0.04 (0) | 0.04 (0) |  |
|  | Median [min, max] | 0.47 [0.04, 3.00] | 0.57 [0.04, 1.90] | 0.132 | 0.04 [0.04, 0.04] | 0.04 [0.04, 0.04] |  |
| LNFP V | |  |  |  |  |  |  |
|  | Mean (SD) | 0.02 (0.01) | 0.02 (0.01) |  | 0.10 (0.04) | 0.09 (0.05) |  |
|  | Median [min, max] | 0.02 [0.01, 0.09] | 0.02 [0.01, 0.05] | 0.331 | 0.10 [0.01, 0.21] | 0.08 [0.03, 0.24] | 0.112 |
| LNFP III | |  |  |  |  |  |  |
|  | Mean (SD) | 0.17 (0.07) | 0.17 (0.07) |  | 0.22 (0.07) | 0.20 (0.08) |  |
|  | Median [min, max] | 0.17 [0.05, 0.48] | 0.17 [0.04, 0.39] | 0.723 | 0.21 [0.08, 0.43] | 0.19 [0.09, 0.43] | 0.099 |
| LNFP II | |  |  |  |  |  |  |
|  | Mean (SD) | 0.19 (0.16) | 0.18 (0.12) |  | 1.01 (0.43) | 0.94 (0.47) |  |
|  | Median [min, max] | 0.14 [0.04, 1.20] | 0.13 [0.04, 0.61] | 0.901 | 0.99 [0.04, 2.00] | 0.84 [0.24, 2.50] | 0.148 |
| LNDFH I | |  |  |  |  |  |  |
|  | Mean (SD) | 0.65 (0.33) | 0.69 (0.33) |  | 0.02 (0) | 0.02 (0) |  |
|  | Median [min, max] | 0.63 [0.02, 1.70] | 0.66 [0.02, 1.90] | 0.247 | 0.02 [0.02, 0.02] | 0.02 [0.02, 0.02] |  |
| LNDFH II + LNnDFH II | |  |  |  |  |  |  |
|  | Mean (SD) | 0.02 (0.02) | 0.02 (0.02) |  | 0.24 (0.12) | 0.24 (0.17) |  |
|  | Median [min, max] | 0.01 [0.01, 0.15] | 0.01 [0.01, 0.18] | 0.645 | 0.23 [0.01, 0.49] | 0.20 [0.04, 0.73] | 0.312 |
| Sum of HMOs | |  |  |  |  |  |  |
|  | Mean (SD) | 6.46 (1.14) | 6.68 (1.24) |  | 5.13 (0.97) | 4.98 (1.30) |  |
|  | Median [min, max] | 6.30 [3.78, 11.6] | 6.56 [3.58, 11.1] | 0.066 | 5.10 [2.41, 7.20] | 4.75 [2.76, 8.40] | 0.232 |

P values derived from Wilcoxon sum-rank test comparing HMO concentrations between infants with and without URTI at 1 year within the secretor and non-secretor milk groups. Bonferroni-adjusted level of statistical significance is α = 0.05/16 = 0.0031. URTI- Upper respiratory tract infections; HMO, human milk oligosaccharides. 2’-FL, 2’-fucosyllactose; 3-FL, 3-fucosyllactose; 3’-SL, 3’-sialyllactose; 6'-GL, 6’-Galactosyllactose; DFL, 3,2’-difucosyllactose; 6’-SL; 6’-sialyllactose; LNT, lacto-N-tetraose; LNnT, lacto-N-neotetraose; LNFP I, lacto-N-fucopentaose-I; LNFP V, lacto-N-fucopentaose-V; LNFP III, lacto-N-fucopentaose-III; LNFP II, lacto-N-fucopentaose-II; LNDFH I, lacto-N-difucohexaose I; LNDFH II, lacto-N-difucohexaose II; LNnDFH II, lacto-N-neodifucohexaose II.

**Table 12**. Absolute human milk oligosaccharide concentrations (g/L) measured at 6 weeks stratified by maternal secretor status and upper respiratory tract infections (URTI) in infants in the second year of life in the Ulm SPATZ Health Study

|  |  | Secretor milk | |  | Non-secretor milk | |  |
| --- | --- | --- | --- | --- | --- | --- | --- |
|  |  | URTI Yes (n=342) | URTI No (n=40) | p | URTI Yes (n=89) | URTI No (n=15) | p |
| Lactose | |  |  |  |  |  |  |
|  | Mean (SD) | 66.6 (3.73) | 66.4 (3.77) |  | 67.2 (3.79) | 67.5 (2.59) |  |
|  | Median [min, max] | 67.0 [52.0, 77.0] | 67.0 [53.0, 74.0] | 0.84 | 68.0 [56.0, 74.0] | 67.0 [63.0, 72.0] | 0.794 |
| 2'-FL | |  |  |  |  |  |  |
|  | Mean (SD) | 2.79 (0.99) | 2.93 (1.04) |  | 0.13 (0) | 0.13 (0) |  |
|  | Median [min, max] | 2.70 [0.13, 6.60] | 2.70 [0.33, 5.60] | 0.361 | 0.13 [0.13, 0.13] | 0.13 [0.13, 0.13] |  |
| 3-FL | |  |  |  |  |  |  |
|  | Mean (SD) | 0.46 (0.27) | 0.43 (0.26) |  | 1.50 (0.55) | 1.49 (0.47) |  |
|  | Median [min, max] | 0.41 [0.03, 1.90] | 0.39 [0.03, 1.30] | 0.41 | 1.50 [0.08, 2.90] | 1.60 [0.63, 2.40] | 0.897 |
| 3'-SL | |  |  |  |  |  |  |
|  | Mean (SD) | 0.14 (0.04) | 0.15 (0.04) |  | 0.18 (0.05) | 0.17 (0.05) |  |
|  | Median [min, max] | 0.14 [0.05, 0.26] | 0.15 [0.08, 0.27] | 0.344 | 0.17 [0.07, 0.32] | 0.16 [0.11, 0.27] | 0.455 |
| 6'-GL | |  |  |  |  |  |  |
|  | Mean (SD) | 0.02 (0.01) | 0.02 (0.01) |  | 0.02 (0.02) | 0.02 (0.01) |  |
|  | Median [min, max] | 0.02 [0.004, 0.09] | 0.02 [0.01, 0.04] | 0.299 | 0.02 [0.01, 0.150] | 0.02 [0.01, 0.03] | 0.223 |
| DFL | |  |  |  |  |  |  |
|  | Mean (SD) | 0.22 (0.16) | 0.22 (0.15) |  | 0.01 (0) | 0.01 (0) |  |
|  | Median [min, max] | 0.20 [0.02, 1.80] | 0.21 [0.01, 0.88] | 0.577 | 0.01 [0.01, 0.01] | 0.01 [0.01, 0.01] |  |
| 6'-SL | |  |  |  |  |  |  |
|  | Mean (SD) | 0.26 (0.11) | 0.26 (0.10) |  | 0.25 (0.10) | 0.25 (0.11) |  |
|  | Median [min, max] | 0.24 [0.05, 0.73] | 0.24 [0.07, 0.54] | 0.683 | 0.24 [0.07, 0.55] | 0.22 [0.13, 0.43] | 0.937 |
| LNT | |  |  |  |  |  |  |
|  | Mean (SD) | 0.83 (0.41) | 0.84 (0.36 |  | 1.36 (0.61) | 1.33 (0.53) |  |
|  | Median [min, max] | 0.76 [0.14, 2.90] | 0.80 [0.19, 1.70] | 0.45 | 1.30 [0.24, 3.10] | 1.30 [0.51, 2.20] | 0.974 |
| LNnT | |  |  |  |  |  |  |
|  | Mean (SD) | 0.10 (0.07) | 0.10 (0.02) |  | 0.03 (0.02) | 0.02 (0.03) |  |
|  | Median [min, max] | 0.09 [0.01, 0.35] | 0.09 [0.02, 0.24] | 0.75 | 0.02 [0.01, 0.10] | 0.02 [0.01, 0.13] | 0.075 |
| LNFP I | |  |  |  |  |  |  |
|  | Mean (SD) | 0.58 (0.43) | 0.70 (0.50) |  | 0.04 (0) | 0.04 (0) |  |
|  | Median [min, max] | 0.50 [0.04, 3.00] | 0.59 [0.04, 2.70] | 0.067 | 0.04 [0.04, 0.04] | 0.04 [0.04, 0.04] |  |
| LNFP V | |  |  |  |  |  |  |
|  | Mean (SD) | 0.02 (0.01) | 0.02 (0.01) |  | 0.09 (0.04) | 0.10 (0.06) |  |
|  | Median [min, max] | 0.02 [0.01, 0.09] | 0.02 [0.01, 0.05] | 0.94 | 0.09 [0.01, 0.21] | 0.09 [0.05, 0.24] | 0.959 |
| LNFP III | |  |  |  |  |  |  |
|  | Mean (SD) | 0.17 (0.07) | 0.17 (0.08) |  | 0.21 (0.07) | 0.21 (0.09) |  |
|  | Median [min, max] | 0.17 [0.04, 0.48] | 0.17[0.05, 0.39] | 0.842 | 0.20 [0.08, 0.4] | 0.19 [0.10, 0.43] | 0.604 |
| LNFP II | |  |  |  |  |  |  |
|  | Mean (SD) | 0.19 (0.16) | 0.18 (0.12) |  | 0.97 (0.46) | 0.94 (0.42) |  |
|  | Median [min, max] | 0.13 [0.04, 1.20] | 0.16 [0.04, 0.47] | 0.933 | 0.95 [0.04, 2.50] | 0.82 [0.39, 1.70] | 0.484 |
| LNDFH I | |  |  |  |  |  |  |
|  | Mean (SD) | 0.66 (0.32) | 0.68 (0.32) |  | 0.02 (0) | 0.02 (0) |  |
|  | Median [min, max] | 0.63 [0.02, 1.90] | 0.66 [0.02, 1.70] | 0.441 | 0.02 [0.02, 0.02] | 0.02 [0.02, 0.02] |  |
| LNDFH II + LNnDFH II | |  |  |  |  |  |  |
|  | Mean (SD) | 0.02 (0.02) | 0.02 (0.02) |  | 0.24 (0.14) | 0.23 (0.17) |  |
|  | Median [min, max] | 0.01 [0.01, 0.18] | 0.01 [0.01, 0.06] | 0.371 | 0.22 [0.01, 0.73] | 0.23 [0.07, 0.70] | 0.604 |
| Sum of HMOs | |  |  |  |  |  |  |
|  | Mean (SD) | 6.46 (1.16) | 6.71 (1.20) |  | 5.04 (1.10) | 4.97 (1.20) |  |
|  | Median [min, max] | 6.31 [3.78, 11.6] | 6.81 [3.58, 9.80] | 0.031 | 4.99 [2.41, 8.40] | 4.69 [3.39, 7.43] | 0.456 |

P values derived from Wilcoxon sum-rank test comparing HMO concentrations between infants with and without URTI at 2 years within the secretor and non-secretor milk groups. Bonferroni-adjusted level of statistical significance is α = 0.05/16 = 0.0031. URTI- Upper respiratory tract infections; HMO, human milk oligosaccharides. 2’-FL, 2’-fucosyllactose; 3-FL, 3-fucosyllactose; 3’-SL, 3’-sialyllactose; 6'-GL, 6’-Galactosyllactose; DFL, 3,2’-difucosyllactose; 6’-SL; 6’-sialyllactose; LNT, lacto-N-tetraose; LNnT, lacto-N-neotetraose; LNFP I, lacto-N-fucopentaose-I; LNFP V, lacto-N-fucopentaose-V; LNFP III, lacto-N-fucopentaose-III; LNFP II, lacto-N-fucopentaose-II; LNDFH I, lacto-N-difucohexaose I; LNDFH II, lacto-N-difucohexaose II; LNnDFH II, lacto-N-neodifucohexaose II.

**Table 13**. Absolute human milk oligosaccharide concentrations (g/L) measured at 6 weeks stratified by milk group and upper respiratory tract infections (URTI) in infants in the first year of life in the Ulm SPATZ Health Study

|  |  | Group I | |  | Group II milk | |  |
| --- | --- | --- | --- | --- | --- | --- | --- |
|  |  | URTI Yes (n=238) | URTI No (n=122) | p | URTI Yes (n=59) | URTI No (n=37) | p |
| Lactose | |  |  |  |  |  |  |
|  | Mean (SD) | 66.6 (3.70) | 66.1 (3.92) |  | 67.6 (3.89) | 66.8 (3.52) |  |
|  | Median [min, max] | 67.0 [53.0, 74.0] | 66.5 [52.0, 77.0] | 0.153 | 69.0 [58.0, 74.0] | 67.0 [56.0, 72.0] | 0.18 |
| 2'-FL | |  |  |  |  |  |  |
|  | Mean (SD) | 2.63 (0.87) | 2.82 (0.96) |  | 0.13 (0) | 0.13 (0) |  |
|  | Median [min, max] | 2.60 [0.31, 6.30] | 2.70 [0.13, 5.60] | 0.063 | 0.13 [0.13, 0.13] | 0.13 [0.13, 0.13] |  |
| 3-FL | |  |  |  |  |  |  |
|  | Mean (SD) | 0.50 (0.25) | 0.46 (0.26) |  | 1.60 (0.44) | 1.51 (0.50) |  |
|  | Median [min, max] | 0.44 [0.09, 1.50] | 0.42 [0.10, 1.90] | 0.113 | 1.70 [0.72, 2.60] | 1.50 [0.63, 2.90] | 0.267 |
| 3'-SL | |  |  |  |  |  |  |
|  | Mean (SD) | 0.15 (0.04) | 0.15 (0.04) |  | 0.18 (0.05) | 0.18 (0.06) |  |
|  | Median [min, max] | 0.14 [0.07, 0.26] | 0.15 [0.08, 0.27] | 0.45 | 0.17 [0.09, 0.30] | 0.17 [0.07, 0.32] | 0.8 |
| 6'-GL | |  |  |  |  |  |  |
|  | Mean (SD) | 0.02 (0.01) | 0.02 (0.01) |  | 0.02 (0.02) | 0.02 (0.01) |  |
|  | Median [min, max] | 0.02 [0.004, 0.05] | 0.02 [0.01, 0.09] | 0.596 | 0.02 [0.01, 0.15] | 0.02 [0.01, 0.03] | 0.129 |
| DFL | |  |  |  |  |  |  |
|  | Mean (SD) | 0.23 (0.13) | 0.25 (0.21) |  | 0.01 (0) | 0.01 (0) |  |
|  | Median [min, max] | 0.21 [0.05, 1.40] | 0.21 [0.05, 1.80] | 0.971 | 0.01 [0.01, 0.01] | 0.01 [0.01, 0.01] |  |
| 6'-SL | |  |  |  |  |  |  |
|  | Mean (SD) | 0.26 (0.10) | 0.26 (0.11) |  | 0.25 (0.09) | 0.25 (0.11) |  |
|  | Median [min, max] | 0.24 [0.07, 0.63] | 0.25 [0.06, 0.61] | 0.557 | 0.23 [0.08, 0.47] | 0.23 [0.07, 0.55] | 0.901 |
| LNT | |  |  |  |  |  |  |
|  | Mean (SD) | 0.85 (0.41) | 0.85 (0.40) |  | 1.34 (0.60) | 1.35 (0.61) |  |
|  | Median [min, max] | 0.81 [0.14, 2.60] | 0.79 [0.14, 2.90] | 0.986 | 1.20 [0.36, 3.10] | 1.30 [0.24, 2.60] | 0.833 |
| LNnT | |  |  |  |  |  |  |
|  | Mean (SD) | 0.10 (0.06) | 0.10 (0.06) |  | 0.03 (0.02) | 0.02 (0.02) |  |
|  | Median [min, max] | 0.09 [0.01, 0.33] | 0.09 [0.02, 0.35] | 0.912 | 0.02 [0.01, 0.10] | 0.02 [0.01, 0.13] | 0.044 |
| LNFP I | |  |  |  |  |  |  |
|  | Mean (SD) | 0.51 (0.34) | 0.63 (0.43) |  | 0.04 (0) | 0.04 (0) |  |
|  | Median [min, max] | 0.43 [0.04, 1.70] | 0.55 [0.04, 1.90] | 0.037 | 0.04 [0.04, 0.04] | 0.04 [0.04, 0.04] |  |
| LNFP V | |  |  |  |  |  |  |
|  | Mean (SD) | 0.02 (0.01) | 0.02 (0.01) |  | 0.10 (0.03) | 0.09 (0.05) |  |
|  | Median [min, max] | 0.02 [0.01, 0.09] | 0.02 [0.01, 0.05] | 0.185 | 0.10 [0.05, 0.17] | 0.08 [0.03, 0.24] | 0.065 |
| LNFP III | |  |  |  |  |  |  |
|  | Mean (SD) | 0.18 (0.07) | 0.18 (0.07) |  | 0.22 (0.07) | 0.20 (0.08) |  |
|  | Median [min, max] | 0.17 [0.05, 0.48] | 0.18 [0.04, 0.39] | 0.927 | 0.21 [0.08, 0.43] | 0.19 [0.09, 0.43] | 0.121 |
| LNFP II | |  |  |  |  |  |  |
|  | Mean (SD) | 0.21 (0.16) | 0.19(0.12) |  | 1.07 (0.36) | 0.94 (0.47) |  |
|  | Median [min, max] | 0.15 [0.04, 1.20] | 0.16 [0.04, 0.61] | 0.421 | 1.00 [0.34, 2.00] | 0.84 [0.24, 2.50] | 0.037 |
| LNDFH I | |  |  |  |  |  |  |
|  | Mean (SD) | 0.71 (0.27) | 0.73 (0.29) |  | 0.02 (0) | 0.02 (0) |  |
|  | Median [min, max] | 0.66 [0.19, 1.70] | 0.68 [0.07, 1.90] | 0.572 | 0.02 [0.02, 0.02] | 0.02 [0.02, 0.02] |  |
| LNDFH II + LNnDFH II | |  |  |  |  |  |  |
|  | Mean (SD) | 0.02 (0.02) | 0.02 (0.02) |  | 0.26(0.11) | 0.24 (0.17) |  |
|  | Median [min, max] | 0.01 [0.01, 0.15] | 0.01 [0.01, 0.18] | 0.376 | 0.25 [0.05, 0.49] | 0.20 [0.04, 0.73] | 0.105 |
| Sum of HMOs | |  |  |  |  |  |  |
|  | Mean (SD) | 6.39 (1.07) | 6.68 (1.24) |  | 5.25 (0.86) | 4.98 (1.30) |  |
|  | Median [min, max] | 6.26 [3.78, 10.7] | 6.56 [3.58, 11.1] | 0.025 | 5.20 [3.27, 7.20] | 4.75 [2.76, 8.40] | 0.088 |

P values derived from Wilcoxon sum-rank test comparing HMO concentrations between infants with and without URTI at 1 year within milk groups I and II. Bonferroni-adjusted level of statistical significance is α = 0.05/16 = 0.0031. URTI- Upper respiratory tract infections; HMO, human milk oligosaccharides. 2’-FL, 2’-fucosyllactose; 3-FL, 3-fucosyllactose; 3’-SL, 3’-sialyllactose; 6'-GL, 6’-Galactosyllactose; DFL, 3,2’-difucosyllactose; 6’-SL; 6’-sialyllactose; LNT, lacto-N-tetraose; LNnT, lacto-N-neotetraose; LNFP I, lacto-N-fucopentaose-I; LNFP V, lacto-N-fucopentaose-V; LNFP III, lacto-N-fucopentaose-III; LNFP II, lacto-N-fucopentaose-II; LNDFH I, lacto-N-difucohexaose I; LNDFH II, lacto-N-difucohexaose II; LNnDFH II, lacto-N-neodifucohexaose II.

**Table 14**. Absolute human milk oligosaccharide concentrations measured at 6 weeks stratified by milk group and upper respiratory tract infections (URTI) in infants in the second year of life in the Ulm SPATZ Health Study

|  |  | Group I milk | |  | Group II | |  |
| --- | --- | --- | --- | --- | --- | --- | --- |
|  |  | URTI Yes (n=316) | URTI No (n=80) | p | URTI Yes (n=84) | URTI No (n=15) | p |
| Lactose | |  |  |  |  |  |  |
|  | Mean (SD) | 66.6 (3.71) | 66.5 (3.69) |  | 67.2 (3.88) | 67.5 (2.59) |  |
|  | Median [min, max] | 67.0 [52.0, 77.0] | 67.0 [53.0, 74.0] | 0.955 | 68.0 [56.0, 74.0] | 67.0 [63.0, 72.0] | 0.841 |
| 2'-FL | |  |  |  |  |  |  |
|  | Mean (SD) | 2.68 (0.88) | 2.80 (0.94) |  | 0.13 (0) | 0.13 (0) |  |
|  | Median [min, max] | 2.60 [0.13, 6.30] | 2.70 [0.33, 5.60] | 0.389 | 0.13 [0.13, 0.13] | 0.13 [0.13, 0.13] |  |
| 3-FL | |  |  |  |  |  |  |
|  | Mean (SD) | 0.49 (0.25) | 0.46 (0.24) |  | 1.57 (0.47) | 1.49 (0.47) |  |
|  | Median [min, max] | 0.43 [0.09, 1.90] | 0.42 [0.10, 1.30] | 0.443 | 1.60 [0.72, 2.90] | 1.60 [0.63, 2.40] | 0.614 |
| 3'-SL | |  |  |  |  |  |  |
|  | Mean (SD) | 0.14 (0.04) | 0.15 (0.04) |  | 0.18 (0.06) | 0.18 (0.05) |  |
|  | Median [min, max] | 0.14 [0.05, 0.26] | 0.15 [0.08, 0.27] | 0.38 | 0.17 [0.07, 0.32] | 0.16 [0.11, 0.27] | 0.406 |
| 6'-GL | |  |  |  |  |  |  |
|  | Mean (SD) | 0.02 (0.01) | 0.02 (0.01) |  | 0.02 (0.02) | 0.02 (0.01) |  |
|  | Median [min, max] | 0.02 [0.004, 0.09] | 0.02 [0.01, 0.03] | 0.72 | 0.02 [0.01, 0.15] | 0.05 [0.01, 0.03] | 0.287 |
| DFL | |  |  |  |  |  |  |
|  | Mean (SD) | 0.24 (0.16) | 0.24 (0.14) |  | 0.01 (0) | 0.01 (0) |  |
|  | Median [min, max] | 0.20 [0.05, 1.80] | 0.215 [0.05, 0.88] | 0.415 | 0.01 [0.01, 0.01] | 0.01 [0.01, 0.01] |  |
| 6'-SL | |  |  |  |  |  |  |
|  | Mean (SD) | 0.26 (0.10) | 0.26 (0.10) |  | 0.25 (0.10) | 0.25 (0.11) |  |
|  | Median [min, max] | 0.23 [0.05, 0.63] | 0.25 [0.07, 0.54] | 0.403 | 0.24 [0.07, 0.55] | 0.22 [0.13, 0.43] | 1 |
| LNT | |  |  |  |  |  |  |
|  | Mean (SD) | 0.84 (0.41) | 0.86 (0.36) |  | 1.33 (0.61) | 1.33 (0.53) |  |
|  | Median [min, max] | 0.79 [0.14, 2.90] | 0.85 [0.19, 1.70] | 0.379 | 1.20 [0.24, 3.10] | 1.30 [0.51, 2.20] | 0.792 |
| LNnT | |  |  |  |  |  |  |
|  | Mean (SD) | 0.10 (0.06) | 0.10 (0.05) |  | 0.03 (0.0194) | 0.02 (0.03) |  |
|  | Median [min, max] | 0.09 [0.01, 0.35] | 0.09 [0.02, 0.24] | 0.63 | 0.02 [0.01, 0.10] | 0.02 [0.01, 0.13] |  |
| LNFP I | |  |  |  |  |  | 0.086 |
|  | Mean (SD) | 0.53 (0.36) | 0.64 (0.44) |  | 0.04 (0) | 0.04 (0) |  |
|  | Median [min, max] | 0.44 [0.04, 1.90] | 0.55 [0.04, 1.80] | 0.066 | 0.04 [0.04, 0.04] | 0.04 [0.04, 0.04] |  |
| LNFP V | |  |  |  |  |  |  |
|  | Mean (SD) | 0.02 (0.01) | 0.02 (0.01) |  | 0.10 (0.04) | 0.10 (0.06) |  |
|  | Median [min, max] | 0.02 [0.01, 0.09] | 0.02 [0.01, 0.05] | 0.894 | 0.01 [0.03, 0.21] | 0.09 [0.05, 0.24] | 0.938 |
| LNFP III | |  |  |  |  |  |  |
|  | Mean (SD) | 0.18 (0.07) | 0.18 (0.08) |  | 0.21 (0.07) | 0.21 (0.08) |  |
|  | Median [min, max] | 0.17 [0.04, 0.48] | 0.17 [0.05, 0.39] | 0.933 | 0.20 [0.08, 0.43] | 0.19 [0.10, 0.43] | 0.642 |
| LNFP II | |  |  |  |  |  |  |
|  | Mean (SD) | 0.20 (0.16) | 0.19 (0.12) |  | 1.02 (0.41) | 0.94 (0.42) |  |
|  | Median [min, max] | 0.14 [0.04, 1.20] | 0.18 [0.04, 0.47] | 0.866 | 0.98 [0.24, 2.50] | 0.82 [0.39, 1.70] | 0.27 |
| LNDFH I | |  |  |  |  |  |  |
|  | Mean (SD) | 0.71 (0.27) | 0.74 (0.27) |  | 0.02 (0) | 0.02 (0) |  |
|  | Median [min, max] | 0.66 [0.07, 1.90] | 0.68 [0.14, 1.70] | 0.347 | 0.02 [0.02, 0.02] | 0.02 [0.02, 0.02] |  |
| LNDFH II + LNnDFH II | |  |  |  |  |  |  |
|  | Mean (SD) | 0.02 (0.02) | 0.02 (0.02) |  | 0.25 (0.13) | 0.23 (0.17) |  |
|  | Median [min, max] | 0.01 [0.01, 0.18] | 0.02 [0.01, 0.06] | 0.358 | 0.23 [0.04, 0.73] | 0.23 [0.07, 0.70] | 0.361 |
| Sum of HMOs | |  |  |  |  |  |  |
|  | Mean (SD) | 6.41 (1.11) | 6.67 (1.15) |  | 5.15 (1.03) | 4.97 (1.20) |  |
|  | Median [min, max] | 6.27 [3.78, 11.1] | 6.75 [3.58, 9.34] | 0.027 | 5.07 [2.76, 8.40] | 4.69 [3.39, 7.43] | 0.262 |

P values derived from Wilcoxon sum-rank test comparing HMO concentrations between infants with and without URTI at 2 years within milk groups I and II. Bonferroni-adjusted level of statistical significance is α = 0.05/16 = 0.0031. URTI- Upper respiratory tract infections; HMO, human milk oligosaccharides. 2’-FL, 2’-fucosyllactose; 3-FL, 3-fucosyllactose; 3’-SL, 3’-sialyllactose; 6'-GL, 6’-Galactosyllactose; DFL, 3,2’-difucosyllactose; 6’-SL; 6’-sialyllactose; LNT, lacto-N-tetraose; LNnT, lacto-N-neotetraose; LNFP I, lacto-N-fucopentaose-I; LNFP V, lacto-N-fucopentaose-V; LNFP III, lacto-N-fucopentaose-III; LNFP II, lacto-N-fucopentaose-II; LNDFH I, lacto-N-difucohexaose I; LNDFH II, lacto-N-difucohexaose II; LNnDFH II, lacto-N-neodifucohexaose II.

**Table 15**. Adjusted associations between human milk oligosaccharides measured at 6 weeks of lactation with lower respiratory tract infections (LRTI) in the first or second year of life in the Ulm SPATZ Health Study

|  | LRTI in the first year of life | | |  | LRTI in the second year of life | | |
| --- | --- | --- | --- | --- | --- | --- | --- |
|  | RR | 95% CI | P value |  | RR | 95% CI | P value |
| Lactose | 0.99 | (0.98, 1.01) | 0.34 |  | 1.00 | (0.99, 1.01) | 0.47 |
| 2'-FL | 1.00 | (0.92, 1.08) | 0.96 |  | 0.98 | (0.92, 1.05) | 0.61 |
| 3-FL | 0.95 | (0.86, 1.05) | 0.34 |  | 1.02 | (0.93, 1.11) | 0.73 |
| 3'-SL | 1.01 | (0.96, 1.07) | 0.64 |  | 1.02 | (0.97, 1.06) | 0.47 |
| 6'-GL | 0.98 | (0.88, 1.09) | 0.67 |  | 0.95 | (0.86, 1.05) | 0.34 |
| DFL | 1.06 | (0.90, 1.25) | 0.47 |  | 0.95 | (0.83, 1.09) | 0.46 |
| 6'-SL | 1.03 | (0.95, 1.12) | 0.41 |  | 1.01 | (0.94, 1.08) | 0.84 |
| LNT | 1.03 | (0.94, 1.14) | 0.51 |  | 1.01 | (0.94, 1.10) | 0.72 |
| LNnT | 0.99 | (0.87, 1.14) | 0.94 |  | 1.03 | (0.91, 1.15) | 0.68 |
| LNFP I | 1.05 | (0.89, 1.24) | 0.56 |  | 1.02 | (0.89, 1.17) | 0.78 |
| LNFP V | 1.02 | (0.91, 1.15) | 0.75 |  | 1.01 | (0.91, 1.12) | 0.90 |
| LNFP III | 1.00 | (0.92, 1.09) | 0.97 |  | 0.95 | (0.89, 1.02) | 0.18 |
| LNFP II | 0.95 | (0.84, 1.08) | 0.47 |  | 1.01 | (0.90, 1.14) | 0.87 |
| LNDFH I | 1.03 | (0.93, 1.16) | 0.55 |  | 1.01 | (0.92, 1.11) | 0.82 |
| LNDFH II + LNnDFH II | 0.88 | (0.73, 1.07) | 0.20 |  | 0.99 | (0.82, 1.19) | 0.91 |
| Total HMOs | 1.00 | (0.96, 1.04) | 0.91 |  | 1.00 | (0.97, 1.03) | 0.91 |

Associations determined by modified Poisson regression. Models adjusted for infant sex, maternal allergy, delivery mode, exclusive breastfeeding, parity, secretor status and milk group. Bonferroni-adjusted level of statistical significance is α = 0.05/16 = 0.0031. LRTI- Lower respiratory tract infections; RR- Risk Ratio; CI- Confidence intervals; 2’-FL, 2’-fucosyllactose; 3-FL, 3-fucosyllactose; 3’-SL, 3’-sialyllactose; 6'-GL, 6’-Galactosyllactose; DFL, 3,2’-difucosyllactose; 6’-SL; 6’-sialyllactose; LNT, lacto-N-tetraose; LNnT, lacto-N-neotetraose; LNFP I, lacto-N-fucopentaose-I; LNFP V, lacto-N-fucopentaose-V; LNFP III, lacto-N-fucopentaose-III; LNFP II, lacto-N-fucopentaose-II; LNDFH I, lacto-N-difucohexaose I; LNDFH II, lacto-N-difucohexaose II; LNnDFH II, lacto-N-neodifucohexaose II; HMO, human milk oligosaccharides

**Table 16**. Adjusted associations between human milk oligosaccharides in secretor and non-secretor milk measured at 6 weeks of lactation with lower respiratory tract infections (LRTI) in the first or second year of life in the Ulm SPATZ Health Study

|  | Secretor milk | | | | | | |  | Non-secretor milk^*^ | | | | | | |
| --- | --- | --- | --- | --- | --- | --- | --- | --- | --- | --- | --- | --- | --- | --- | --- |
|  | LRTI in the first year of life | | |  | LRTI in the second year of life | | |  | LRTI in the first year of life | | |  | LRTI in the second year of life | | |
|  | RR | 95% CI | p value |  | RR | 95% CI | p value |  | RR | 95% CI | p value |  | RR | 95% CI | p value |
| Lactose | 0.99 | (0.98, 1.01) | 0.32 |  | 1.00 | (0.99, 1.01) | 0.59 |  | 1.00 | (0.98, 1.02) | 0.97 |  | 1.00 | (0.98, 1.02) | 0.99 |
| 2'-FL | 0.99 | (0.92, 1.07) | 0.83 |  | 0.97 | (0.91, 1.03) | 0.34 |  |  |  |  |  |  |  |  |
| 3-FL | 0.99 | (0.89, 1.11) | 0.90 |  | 1.02 | (0.92, 1.13) | 0.76 |  | 0.89 | (0.78, 1.01) | 0.08 |  | 0.98 | (0.87, 1.11) | 0.81 |
| 3'-SL | 1.00 | (0.95, 1.06) | 0.89 |  | 0.98 | (0.93, 1.03) | 0.45 |  | 1.03 | (0.91, 1.16) | 0.63 |  | 1.13 | (1.01, 1.27) | 0.04 |
| 6'-GL | 0.99 | (0.90, 1.10) | 0.86 |  | 0.95 | (0.87, 1.04) | 0.26 |  | 0.96 | (0.75, 1.23) | 0.75 |  | 0.95 | (0.72, 1.25) | 0.73 |
| DFL | 1.07 | (0.91, 1.25) | 0.41 |  | 0.97 | (0.85, 1.10) | 0.60 |  |  |  |  |  |  |  |  |
| 6'-SL | 1.02 | (0.92, 1.12) | 0.74 |  | 0.98 | (0.90, 1.06) | 0.58 |  | 1.08 | (0.94, 1.26) | 0.28 |  | 1.10 | (0.95, 1.28) | 0.19 |
| LNT | 1.02 | (0.91, 1.13) | 0.77 |  | 1.02 | (0.94, 1.12) | 0.59 |  | 1.08 | (0.91, 1.30) | 0.38 |  | 1.01 | (0.85, 1.20) | 0.90 |
| LNnT | 0.99 | (0.86, 1.15) | 0.91 |  | 1.03 | (0.91, 1.16) | 0.67 |  | 1.14 | (0.81, 1.60) | 0.44 |  | 1.21 | (0.90, 1.62) | 0.21 |
| LNFP I | 1.04 | (0.90, 1.20) | 0.64 |  | 0.99 | (0.87, 1.13) | 0.90 |  |  |  |  |  |  |  |  |
| LNFP V | 1.02 | (0.89, 1.16) | 0.81 |  | 1.03 | (0.93, 1.15) | 0.56 |  | 1.01 | (0.85, 1.19) | 0.95 |  | 0.96 | (0.81, 1.13) | 0.61 |
| LNFP III | 1.00 | (0.91, 1.11) | 0.93 |  | 0.96 | (0.88, 1.04) | 0.31 |  | 0.99 | (0.86, 1.15) | 0.92 |  | 0.96 | (0.84, 1.10) | 0.60 |
| LNFP II | 0.98 | (0.83, 1.16) | 0.82 |  | 1.03 | (0.89, 1.20) | 0.65 |  | 0.90 | (0.77, 1.06) | 0.23 |  | 0.93 | (0.80, 1.10) | 0.41 |
| LNDFH I | 1.04 | (0.96, 1.14) | 0.33 |  | 1.04 | (0.96, 1.12) | 0.38 |  |  |  |  |  |  |  |  |
| LNDFH II + LNnDFH II | 0.99 | (0.83, 1.17) | 0.90 |  | 1.06 | (0.88, 1.27) | 0.53 |  | 0.83 | (0.66, 1.04) | 0.10 |  | 0.90 | (0.72, 1.14) | 0.39 |
| Total HMOs | 1.01 | (0.97, 1.05) | 0.65 |  | 0.99 | (0.96, 1.03) | 0.67 |  | 0.96 | (0.89, 1.04) | 0.37 |  | 0.99 | (0.91, 1.07) | 0.77 |

*Some human milk oligosaccharide structures are not present in non-secretor milk. Associations determined by modified Poisson regression. Models adjusted for infant sex, maternal allergy, delivery mode, exclusive breastfeeding, parity and milk group. Bonferroni-adjusted level of statistical significance is α = 0.05/16 = 0.0031. LRTI- Lower respiratory tract infections; RR- Risk Ratio; CI- Confidence intervals; 2’-FL, 2’-fucosyllactose; 3-FL, 3-fucosyllactose; 3’-SL, 3’-sialyllactose; 6'-GL, 6’-Galactosyllactose; DFL, 3,2’-difucosyllactose; 6’-SL; 6’-sialyllactose; LNT, lacto-N-tetraose; LNnT, lacto-N-neotetraose; LNFP I, lacto-N-fucopentaose-I; LNFP V, lacto-N-fucopentaose-V; LNFP III, lacto-N-fucopentaose-III; LNFP II, lacto-N-fucopentaose-II; LNDFH I, lacto-N-difucohexaose I; LNDFH II, lacto-N-difucohexaose II; LNnDFH II, lacto-N-neodifucohexaose II; HMO, human milk oligosaccharides.

**Table 17**. Adjusted associations between human milk oligosaccharides in group I and group II milk measured at 6 weeks of lactation with lower respiratory tract infections (LRTI) in the first or second year of life in the Ulm SPATZ Health Study

|  | Group I milk | | | | | | |  | Group II milk* | | | | | | |
| --- | --- | --- | --- | --- | --- | --- | --- | --- | --- | --- | --- | --- | --- | --- | --- |
|  | LRTI in the first year of life | | |  | LRTI in the second year of life | | |  | LRTI in the first year of life | | |  | LRTI in the second year of life | | |
|  | RR | 95% CI | P value |  | RR | 95% CI | P value |  | RR | 95% CI | P value |  | RR | 95% CI | P value |
| Lactose | 0.99 | (0.98, 1.01) | 0.49 |  | 1.00 | (0.99, 1.01) | 0.74 |  | 1.00 | (0.98, 1.02) | 0.86 |  | 1.00 | (0.98, 1.02) | 0.88 |
| 2'-FL | 0.99 | (0.91, 1.07) | 0.79 |  | 0.97 | (0.91, 1.04) | 0.47 |  |  |  |  |  |  |  |  |
| 3-FL | 0.99 | (0.89, 1.11) | 0.88 |  | 1.02 | (0.92, 1.13) | 0.75 |  | 0.89 | (0.78, 1.02) | 0.09 |  | 0.99 | (0.88, 1.12) | 0.86 |
| 3'-SL | 1.02 | (0.97, 1.08) | 0.59 |  | 0.99 | (0.94, 1.04) | 0.74 |  | 1.05 | (0.94, 1.18) | 0.39 |  | 1.16 | (1.03, 1.29) | 0.01 |
| 6'-GL | 0.99 | (0.89, 1.10) | 0.82 |  | 0.95 | (0.87, 1.04) | 0.30 |  | 0.95 | (0.73, 1.23) | 0.69 |  | 0.95 | (0.70, 1.27) | 0.72 |
| DFL | 1.06 | (0.90, 1.25) | 0.46 |  | 0.97 | (0.84, 1.10) | 0.61 |  |  |  |  |  |  |  |  |
| 6'-SL | 1.03 | (0.94, 1.14) | 0.52 |  | 0.98 | (0.90, 1.06) | 0.61 |  | 1.08 | (0.93, 1.26) | 0.30 |  | 1.10 | (0.95, 1.28) | 0.22 |
| LNT | 1.03 | (0.92, 1.15) | 0.64 |  | 1.04 | (0.95, 1.14) | 0.43 |  | 1.10 | (0.91, 1.33) | 0.31 |  | 1.02 | (0.85, 1.22) | 0.82 |
| LNnT | 1.01 | (0.87, 1.17) | 0.92 |  | 1.04 | (0.92, 1.19) | 0.52 |  | 1.08 | (0.76, 1.53) | 0.65 |  | 1.14 | (0.85, 1.52) | 0.39 |
| LNFP I | 1.04 | (0.88, 1.21) | 0.67 |  | 1.05 | (0.91, 1.20) | 0.51 |  |  |  |  |  |  |  |  |
| LNFP V | 1.01 | (0.88, 1.17) | 0.84 |  | 1.04 | (0.92, 1.16) | 0.55 |  | 1.04 | (0.88, 1.22) | 0.68 |  | 0.98 | (0.83, 1.15) | 0.80 |
| LNFP III | 1.00 | (0.90, 1.11) | 0.95 |  | 0.96 | (0.88, 1.05) | 0.37 |  | 1.02 | (0.88, 1.18) | 0.80 |  | 0.99 | (0.86, 1.13) | 0.84 |
| LNFP II | 0.98 | (0.82, 1.16) | 0.8 |  | 1.03 | (0.89, 1.20) | 0.66 |  | 0.90 | (0.77, 1.06) | 0.23 |  | 0.93 | (0.79, 1.10) | 0.41 |
| LNDFH I | 1.04 | (0.96, 1.14) | 0.33 |  | 1.04 | (0.96, 1.12) | 0.38 |  |  |  |  |  |  |  |  |
| LNDFH II + LNnDFH II | 0.99 | (0.82, 1.18) | 0.87 |  | 1.06 | (0.88, 1.28) | 0.55 |  | 0.83 | (0.66, 1.04) | 0.10 |  | 0.90 | (0.72, 1.14) | 0.39 |
| Total HMOs | 1.01 | (0.97, 1.05) | 0.67 |  | 1.00 | (0.97, 1.04) | 0.92 |  | 0.97 | (0.90, 1.05) | 0.47 |  | 0.99 | (0.91, 1.08) | 0.87 |

*Some human milk oligosaccharide structures are not present in group II milk. Associations determined by modified Poisson regression. Models adjusted for infant sex, maternal allergy, delivery mode, exclusive breastfeeding, parity and milk group. Bonferroni-adjusted level of statistical significance is α = 0.05/16 = 0.0031. LRTI- Lower respiratory tract infections; RR- Risk Ratio; CI- Confidence intervals; 2’-FL, 2’-fucosyllactose; 3-FL, 3-fucosyllactose; 3’-SL, 3’-sialyllactose; 6'-GL, 6’-Galactosyllactose; DFL, 3,2’-difucosyllactose; 6’-SL; 6’-sialyllactose; LNT, lacto-N-tetraose; LNnT, lacto-N-neotetraose; LNFP I, lacto-N-fucopentaose-I; LNFP V, lacto-N-fucopentaose-V; LNFP III, lacto-N-fucopentaose-III; LNFP II, lacto-N-fucopentaose-II; LNDFH I, lacto-N-difucohexaose I; LNDFH II, lacto-N-difucohexaose II; LNnDFH II, lacto-N-neodifucohexaose II; HMO, human milk oligosaccharides.

**Table 18.** Adjusted associations between human milk oligosaccharides measured at 6 weeks of lactation with upper respiratory tract infections (URTI) in the first or second year of life in the Ulm SPATZ Health Study

|  | URTI in the first year of life | | |  | URTI in the second year of life | | |
| --- | --- | --- | --- | --- | --- | --- | --- |
|  | RR | 95% CI | P value |  | RR | 95% CI | P value |
| Lactose | 1.01 | (1.00, 1.01) | 0.19 |  | 1.00 | (0.99, 1.01) | 0.77 |
| 2'-FL | 0.97 | (0.90, 1.04) | 0.38 |  | 0.96 | (0.89, 1.04) | 0.34 |
| 3-FL | 1.02 | (0.93, 1.11) | 0.67 |  | 1.04 | (0.93, 1.16) | 0.51 |
| 3'-SL | 1.00 | (0.95, 1.04) | 0.92 |  | 0.99 | (0.94, 1.05) | 0.73 |
| 6'-GL | 1.02 | (0.92, 1.12) | 0.74 |  | 1.03 | (0.94, 1.13) | 0.50 |
| DFL | 0.92 | (0.78, 1.07) | 0.26 |  | 1.02 | (0.87, 1.19) | 0.85 |
| 6'-SL | 1.01 | (0.94, 1.09) | 0.74 |  | 1.00 | (0.92, 1.08) | 0.92 |
| LNT | 1.01 | (0.93, 1.11) | 0.75 |  | 1.00 | (0.91, 1.09) | 0.93 |
| LNnT | 0.98 | (0.86, 1.10) | 0.70 |  | 1.03 | (0.91, 1.16) | 0.67 |
| LNFP I | 0.92 | (0.80, 1.05) | 0.24 |  | 0.86 | (0.73, 1.02) | 0.08 |
| LNFP V | 1.09 | (0.99, 1.23) | 0.15 |  | 0.99 | (0.85, 1.16) | 0.94 |
| LNFP III | 1.01 | (0.94, 1.09) | 0.77 |  | 1.01 | (0.93, 1.11) | 0.78 |
| LNFP II | 1.08 | (0.95, 1.23) | 0.24 |  | 1.05 | (0.91, 1.22) | 0.51 |
| LNDFH I | 0.94 | (0.85, 1.03) | 0.22 |  | 0.98 | (0.87, 1.09) | 0.67 |
| LNDFH II + LNnDFH II | 1.03 | (0.85, 1.25) | 0.75 |  | 1.04 | (0.80, 1.34) | 0.79 |
| Total HMOs | 0.98 | (0.95, 1.02) | 0.32 |  | 0.97 | (0.94, 1.01) | 0.21 |

Associations determined by modified Poisson regression. Models adjusted for infant sex, maternal allergy, delivery mode, exclusive breastfeeding, parity, secretor status and milk group. Bonferroni-adjusted level of statistical significance is α = 0.05/16 = 0.0031. URTI- Upper respiratory tract infections; RR- Risk Ratio; CI- Confidence intervals; 2’-FL, 2’-fucosyllactose; 3-FL, 3-fucosyllactose; 3’-SL, 3’-sialyllactose; 6'-GL, 6’-Galactosyllactose; DFL, 3,2’-difucosyllactose; 6’-SL; 6’-sialyllactose; LNT, lacto-N-tetraose; LNnT, lacto-N-neotetraose; LNFP I, lacto-N-fucopentaose-I; LNFP V, lacto-N-fucopentaose-V; LNFP III, lacto-N-fucopentaose-III; LNFP II, lacto-N-fucopentaose-II; LNDFH I, lacto-N-difucohexaose I; LNDFH II, lacto-N-difucohexaose II; LNnDFH II, lacto-N-neodifucohexaose II; HMO, human milk oligosaccharides.

**Table 19**. Adjusted associations between human milk oligosaccharides in secretor and non-secretor milk measured at 6 weeks of lactation with upper respiratory tract infections (URTI) in the first or second year of life in the Ulm SPATZ Health Study

|  | Secretor milk | | | | | | |  | Non-Secretor milk* | | | | | | |
| --- | --- | --- | --- | --- | --- | --- | --- | --- | --- | --- | --- | --- | --- | --- | --- |
|  | URTI in the first year of life | | |  | URTI in the second year of life | | |  | URTI in the first year of life | | |  | URTI in the second year of life | | |
|  | RR | 95% CI | P value |  | RR | 95% CI | P value |  | RR | 95% CI | P value |  | RR | 95% CI | P value |
| Lactose | 1.01 | (0.99, 1.02) | 0.37 |  | 1.00 | (0.99, 1.02) | 0.74 |  | 1.01 | (0.99, 1.03) | 0.43 |  | 0.99 | (0.97, 1.02) | 0.57 |
| 2'-FL | 0.94 | (0.88, 1.01) | 0.09 |  | 0.96 | (0.89, 1.03) | 0.29 |  |  |  |  |  |  |  |  |
| 3-FL | 1.06 | (0.95, 1.19) | 0.28 |  | 1.05 | (0.93, 1.19) | 0.40 |  | 1.07 | (0.94, 1.21) | 0.32 |  | 1.06 | (0.89, 1.26) | 0.51 |
| 3'-SL | 1.00 | (0.95, 1.05) | 0.93 |  | 0.98 | (0.92, 1.04) | 0.45 |  | 1.00 | (0.88, 1.14) | 0.95 |  | 1.06 | (0.91, 1.23) | 0.46 |
| 6'-GL | 0.95 | (0.86, 1.06) | 0.36 |  | 1.00 | (0.91, 1.11) | 0.96 |  | 1.32 | (1.02, 1.70) | 0.03 |  | 1.28 | (0.98, 1.67) | 0.07 |
| DFL | 0.95 | (0.82, 1.10) | 0.47 |  | 1.02 | (0.88, 1.18) | 0.82 |  |  |  |  |  |  |  |  |
| 6'-SL | 1.01 | (0.92, 1.09) | 0.90 |  | 1.00 | (0.91, 1.09) | 0.98 |  | 1.01 | (0.86, 1.19) | 0.93 |  | 1.00 | (0.81, 1.25) | 0.97 |
| LNT | 1.02 | (0.92, 1.13) | 0.68 |  | 0.99 | (0.90, 1.10) | 0.88 |  | 1.01 | (0.84, 1.21) | 0.93 |  | 1.02 | (0.81, 1.29) | 0.85 |
| LNnT | 0.98 | (0.86, 1.11) | 0.71 |  | 1.03 | (0.90, 1.17) | 0.71 |  | 1.22 | (0.85, 1.74) | 0.28 |  | 1.04 | (0.55, 1.96) | 0.90 |
| LNFP I | 0.87 | (0.76, 0.99) | 0.04 |  | 0.86 | (0.73, 1.00) | 0.05 |  |  |  |  |  |  |  |  |
| LNFP V | 1.12 | (1.00, 1.25) | 0.06 |  | 1.05 | (0.93, 1.18) | 0.44 |  | 1.12 | (0.92, 1.36) | 0.25 |  | 0.98 | (0.73, 1.31) | 0.88 |
| LNFP III | 1.00 | (0.91, 1.09) | 0.95 |  | 1.01 | (0.92, 1.12) | 0.81 |  | 1.11 | (0.96, 1.27) | 0.16 |  | 1.01 | (0.83, 1.24) | 0.90 |
| LNFP II | 1.10 | (0.95, 1.27) | 0.20 |  | 1.05 | (0.90, 1.23) | 0.52 |  | 1.17 | (0.97, 1.40) | 0.10 |  | 1.14 | (0.89, 1.45) | 0.30 |
| LNDFH I | 0.99 | (0.91, 1.07) | 0.75 |  | 0.98 | (0.90, 1.07) | 0.62 |  |  |  |  |  |  |  |  |
| LNDFH II + LNnDFH II | 1.06 | (0.88, 1.28) | 0.52 |  | 1.05 | (0.89, 1.23) | 0.58 |  | 1.09 | (0.86, 1.39) | 0.48 |  | 1.11 | (0.77, 1.59) | 0.58 |
| Total HMOs | 0.97 | (0.94, 1.01) | 0.10 |  | 0.97 | (0.93, 1.01) | 0.15 |  | 1.07 | (0.97, 1.17) | 0.17 |  | 1.06 | (0.93, 1.20) | 0.39 |

*Some human milk oligosaccharide structures are not present in non-secretor milk. Associations determined by modified Poisson regression. Models adjusted for infant sex, maternal allergy, delivery mode, exclusive breastfeeding, parity and milk group. Bonferroni-adjusted level of statistical significance is α = 0.05/16 = 0.0031. URTI- Upper respiratory tract infections; RR- Risk Ratio; CI- Confidence intervals; 2’-FL, 2’-fucosyllactose; 3-FL, 3-fucosyllactose; 3’-SL, 3’-sialyllactose; 6'-GL, 6’-Galactosyllactose; DFL, 3,2’-difucosyllactose; 6’-SL; 6’-sialyllactose; LNT, lacto-N-tetraose; LNnT, lacto-N-neotetraose; LNFP I, lacto-N-fucopentaose-I; LNFP V, lacto-N-fucopentaose-V; LNFP III, lacto-N-fucopentaose-III; LNFP II, lacto-N-fucopentaose-II; LNDFH I, lacto-N-difucohexaose I; LNDFH II, lacto-N-difucohexaose II; LNnDFH II, lacto-N-neodifucohexaose II; HMO, human milk oligosaccharides.

**Table 20**. Adjusted associations between human milk oligosaccharides in group I milk measured at 6 weeks of lactation with upper respiratory tract infections (URTI) in the first or second year of life in the Ulm SPATZ Health Study

|  | Group I milk | | | | | | |  | Group II milk* | | | | | | |
| --- | --- | --- | --- | --- | --- | --- | --- | --- | --- | --- | --- | --- | --- | --- | --- |
|  | URTI in the first year of life | | |  | URTI in the second year of life | | |  | URTI in the first year of life | | |  | URTI in the second year of life | | |
|  | RR | 95% CI | P value |  | RR | 95% CI | P value |  | RR | 95% CI | P value |  | RR | 95% CI | P value |
| Lactose | 1.01 | (0.99, 1.02) | 0.30 |  | 1.00 | (0.99, 1.01) | 0.89 |  | 1.01 | (0.99, 1.03) | 0.42 |  | 0.99 | (0.97, 1.02) | 0.57 |
| 2'-FL | 0.94 | (0.87, 1.01) | 0.08 |  | 0.96 | (0.87, 1.04) | 0.33 |  |  |  |  |  |  |  |  |
| 3-FL | 1.06 | (0.95, 1.19) | 0.32 |  | 1.05 | (0.93, 1.19) | 0.46 |  | 1.07 | (0.94, 1.21) | 0.33 |  | 1.06 | (0.89, 1.25) | 0.52 |
| 3'-SL | 1.00 | (0.94, 1.05) | 0.86 |  | 0.98 | (0.92, 1.04) | 0.53 |  | 1.00 | (0.88, 1.14) | 0.98 |  | 1.05 | (0.91, 1.23) | 0.49 |
| 6'-GL | 0.97 | (0.87, 1.08) | 0.59 |  | 1.05 | (0.95, 1.16) | 0.35 |  | 1.32 | (1.02, 1.71) | 0.03 |  | 1.28 | (0.98, 1.67) | 0.07 |
| DFL | 0.93 | (0.80, 1.08) | 0.36 |  | 1.01 | (0.87, 1.17) | 0.92 |  |  |  |  |  |  |  |  |
| 6'-SL | 0.98 | (0.90, 1.07) | 0.63 |  | 0.97 | (0.89, 1.06) | 0.54 |  | 1.01 | (0.86, 1.19) | 0.94 |  | 1.00 | (0.80, 1.25) | 0.98 |
| LNT | 1.01 | (0.91, 1.12) | 0.83 |  | 0.98 | (0.88, 1.09) | 0.72 |  | 1.01 | (0.84, 1.21) | 0.94 |  | 1.02 | (0.81, 1.29) | 0.86 |
| LNnT | 1.02 | (0.89, 1.16) | 0.80 |  | 1.07 | (0.94, 1.23) | 0.29 |  | 1.23 | (0.86, 1.75) | 0.26 |  | 1.06 | (0.57, 1.98) | 0.86 |
| LNFP I | 0.83 | (0.71, 0.95) | 0.01 |  | 0.84 | (0.71, 0.98) | 0.03 |  |  |  |  |  |  |  |  |
| LNFP V | 1.12 | (0.99, 1.26) | 0.07 |  | 1.05 | (0.92, 1.19) | 0.48 |  | 1.11 | (0.92, 1.35) | 0.27 |  | 0.97 | (0.72, 1.30) | 0.83 |
| LNFP III | 0.99 | (0.91, 1.09) | 0.89 |  | 1.01 | (0.91, 1.12) | 0.87 |  | 1.10 | (0.96, 1.27) | 0.17 |  | 1.01 | (0.82, 1.24) | 0.93 |
| LNFP II | 1.10 | (0.95, 1.28) | 0.20 |  | 1.05 | (0.90, 1.24) | 0.51 |  | 1.17 | (0.97, 1.40) | 0.10 |  | 1.14 | (0.89, 1.45) | 0.30 |
| LNDFH I | 0.99 | (0.91, 1.07) | 0.75 |  | 0.98 | (0.90, 1.07) | 0.62 |  |  |  |  |  |  |  |  |
| LNDFH II + LNnDFH II | 1.06 | (0.88, 1.29) | 0.52 |  | 1.05 | (0.89, 1.24) | 0.58 |  | 1.09 | (0.86, 1.38) | 0.48 |  | 1.11 | (0.77, 1.59) | 0.58 |
| Total HMOs | 0.96 | (0.93, 1.00) | 0.04 |  | 0.97 | (0.93, 1.01) | 0.13 |  | 1.07 | (0.97, 1.17) | 0.17 |  | 1.06 | (0.93, 1.20) | 0.40 |

*Some human milk oligosaccharide structures are not present in group II milk. Associations determined by modified Poisson regression. Models adjusted for infant sex, maternal allergy, delivery mode, exclusive breastfeeding, parity and milk group. Bonferroni-adjusted level of statistical significance is α = 0.05/16 = 0.0031. URTI- Upper respiratory tract infections; RR- Risk Ratio; CI- Confidence intervals; 2’-FL, 2’-fucosyllactose; 3-FL, 3-fucosyllactose; 3’-SL, 3’-sialyllactose; 6'-GL, 6’-Galactosyllactose; DFL, 3,2’-difucosyllactose; 6’-SL; 6’-sialyllactose; LNT, lacto-N-tetraose; LNnT, lacto-N-neotetraose; LNFP I, lacto-N-fucopentaose-I; LNFP V, lacto-N-fucopentaose-V; LNFP III, lacto-N-fucopentaose-III; LNFP II, lacto-N-fucopentaose-II; LNDFH I, lacto-N-difucohexaose I; LNDFH II, lacto-N-difucohexaose II; LNnDFH II, lacto-N-neodifucohexaose II; HMO, human milk oligosaccharides.
